# Unravelling the Phytochemical Composition and Antioxidant Potential of Different Parts of *Rumex vesicarius* L.: A RP-HPLC-MS-MS/MS, Chemometrics, and Molecular Docking-Based Comparative Study

**DOI:** 10.3390/plants13131815

**Published:** 2024-07-01

**Authors:** Sherouk Hussein Sweilam, Mohamed S. Abd El Hafeez, Mahmoud A. Mansour, Reham Hassan Mekky

**Affiliations:** 1 Department of Pharmacognosy, College of Pharmacy, Prince Sattam Bin Abdulaziz University, Al-Kharj 11942, Saudi Arabia; 2 Department of Pharmacognosy, Faculty of Pharmacy, Egyptian Russian University, Cairo-Suez Road, Badr City 11829, Cairo, Egypt; mohamed-sayed@eru.edu.eg (M.S.A.E.H.); mahmoud.aly@eru.edu.eg (M.A.M.)

**Keywords:** *Rumex vesicarius*, hydroxycinnamic acids, flavonoids, antioxidant activity, RP-HPLC-MS-MS/MS, PCA, HCA

## Abstract

*Rumex vesicarius* L. Polygonaceae is a wildly grown plant in Egypt, North Africa, and Asia with wide traditional uses. Several studies reported its biological activities and richness in phytochemicals. This research addresses a comprehensive metabolic profiling of the flowers, leaves, stems, and roots via RP-HPLC-QTOF-MS and MS/MS with chemometrics. A total of 60 metabolites were observed and grouped into phenolic acids, flavonoids, phenols, terpenes, amino acids, fatty acids, organic acids, and sugars. Principal component analysis and hierarchal cluster analysis showed the segregation of different parts. Moreover, the antioxidant capacity was determined via several methods and agreed with the previous results. Additionally, an in silico approach of molecular docking of the predominant bioactive metabolites was employed against two antioxidant targets, NADPH oxidase and human peroxiredoxin 5 enzyme (PDB ID: 2CDU and 1HD2) receptors, alongside ADME predictions. The molecular modelling revealed that most of the approached molecules were specifically binding with the tested enzymes, achieving high binding affinities. The results confirmed that *R. vesicarius* stems and roots are rich sources of bioactive antioxidant components. To our knowledge, this is the first comprehensive metabolic profiling of *R. vesicarius* giving a prospect of its relevance in the development of new naturally based antioxidants.

## 1. Introduction

Numerous researchers have focused on exploring new vegetable sources of natural antioxidants for preserving health in response to the increasing need for a healthy lifestyle. Currently, medicinal plants have the potential to be a viable substitute for commercial pharmaceuticals in medicine [1,2,3]. Additionally, natural plant-based substances have better pharmacological activity, are less expensive and more effective, and have fewer adverse effects [4,5]. Furthermore, investigating the novel functions that the chemical compositions of medicinal plants could have in enhancing or improving the qualities of products derived from plants is still necessary in the research of these compositions [6]. Phenolic compounds are one of the main classes of secondary metabolites made by plants. They act as antioxidants and exert powerful protection on food from oxidative damage [7]. In recent years, there has been a surge in interest in natural antioxidants, particularly phenolic compounds from plants.

The family Polygonaceae includes several plants rich in diverse phenolic compounds. Approximately 50 genera and 1200 species belong to this family [8]. *Calligonum*, *Coccoloba*, *Persicaria*, *Polygonum*, *Rheum*, and *Rumex* are considered the largest genera of this family [8]. In Egypt, 28 species in 8 genera represent this family [9]. *Rumex* L. is a prevalent genus found in the northern temperate zone and tropical regions. The genus includes more than 200 species [10]. *Rumex* species exert an important role as alternative medicine, as these plants have consistently been utilized for their medicinal properties in the treatment of various ailments [11]. *R. vesicarius* has been used traditionally for relieving gastrointestinal problems such as flatulence, constipation, and dyspepsia. It has also been used as a tonic and shows analgesic activity. Furthermore, it is used in the treatment of liver and spleen disorders [12]. Reported biological activities of *Rumex* species include anticancer, analgesic, antimicrobial, antidiarrheal, anti-inflammatory, antioxidant, and anthelminthic activities [10,11,13]. These activities are believed to be derived from their complex phytochemical composition, which includes, for instance, ferulic acid, diosmetin, catechins, apigenin, luteolin glycosides, vitamin C, and unsaturated fatty acids which have been identified in *R. vesicarius* leaves. Moreover, several monoterpenes and sesquiterpenes have been identified in the volatile oil of its fruit. Nepodin, chrysophanol, physcion, and emodin have been isolated from *R. vesicarius* root [7,9,10,14,15].

*Rumex vesicarius* L. is a green leafy herb that grows to a height of 15–30 cm. This plant is native to Asia and northern Africa and grows every year during autumn and spring [16]. Numerous studies have demonstrated that its higher concentrations of β-carotenes, vitamins, lipids, proteins, and organic acids make it an excellent nutritional supplement plant [17]. Furthermore, it is rich in bioactive compounds, including flavonoids, anthraquinones, tannins, and mucilage [15,18,19]. Several studies report that *R. vesicarius* is indicated as antioxidant, antihypertensive, anticancer, and antimicrobial, among other activities [8,15,16,19,20].

Several studies addressed the phytochemical composition and biological potentials of different fractions from several plant organs of *R. vesicarius* [16,19]. It showed significant biological activities against tumours, hepatic diseases, cardiovascular diseases, and gastrointestinal disorders, among other ailments [16].

To our knowledge, there are no comprehensive and detailed studies available concerning the metabolic profiling of *R. vesicarius* with an insight into the difference in phytochemical constituents of different parts of *R. vesicarius*. Therefore, the primary goal of this study is to investigate the chemical composition of *R. vesicarius* of different parts, *viz.*, flowers, leaves, roots, and stems, extracts using reversed-phase (RP) high-performance liquid chromatography (HPLC), electrospray ionization (ESI), quadrupole time of flight (QTOF) mass spectrometry (MS) and tandem MS/MS, and chemometrics. Furthermore, the work aimed to explore the antioxidant properties of different parts of the plant. Moreover, in silico molecular docking and ADME investigations were conducted on the predominant components in *R. vesicarius* different parts against antioxidant molecular targets.

## 2. Results and Discussion

### 2.1. Phytochemical Composition of Rumex vesicarius L. Wildly Grown in Egypt

**Figure 1:**
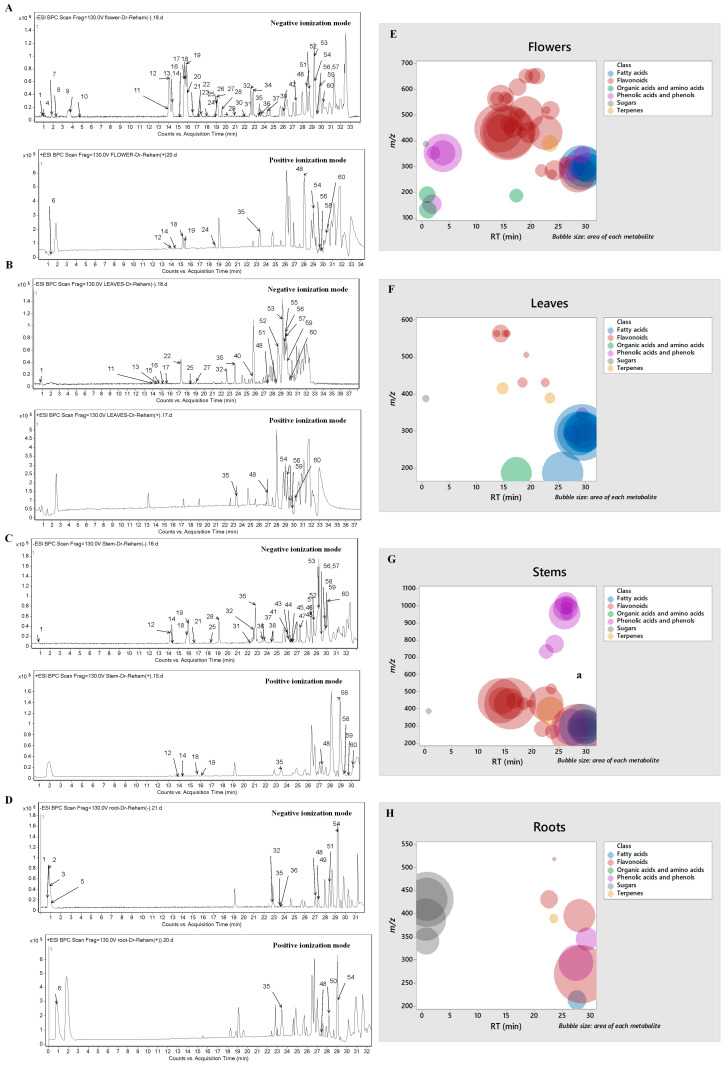
Base peak chromatograms (BPCs) of (**A**) flowers, (**B**) leaves, (**C**) stems, and (**D**) roots of *R. vesicarius* and bubble plot of the observed masses *m*/*z* vs. the retention time concerning metabolite classes and peak areas for the (**E**) flowers, (**F**) leaves, (**G**) stems, and (**H**) roots.

**Figure 2:**
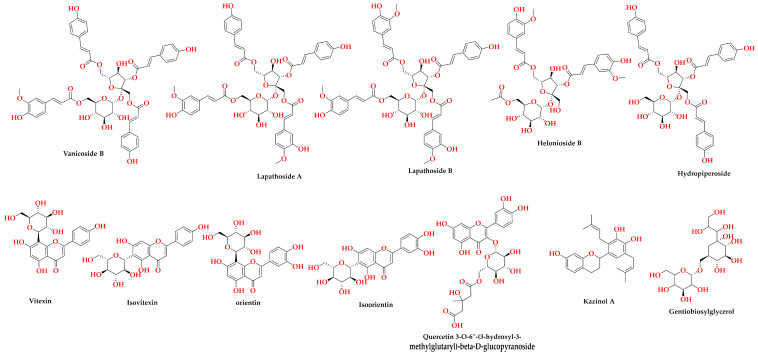
Structures of the major bioactive metabolites in the different parts of *R. vesicarius* L.

The phytochemical composition of the different parts of *R. vesicarius* was performed via RP-HPLC-ESI-QTOF-MS-andMS/MS in both negative and positive ionization modes outlying the occurrence of 60 metabolites distributed in all parts. In this sense, Figure 1A–D show the base peak chromatograms (BPC) of the flowers, leaves, stems, and roots, respectively. Also, Figure 1E,F exhibit the bubble plot of the detected masses *m*/*z* vs. retention time regarding metabolite classes and peak areas for the flowers, leaves, stems, and roots, respectively. It highlights that the elution order depended on the class, and so the basic chemical structure of the compounds, i.e., more polar and small compounds (sugars, organic acids, and amino acids) were eluted first, followed by phenolic acids, ending with flavonoids, and finally the elution of fatty acids (Table 1). The annotated metabolites were classified as flavonoids (27), phenolic acids and other phenols (14), terpenes (2), fatty acids (10), sugars (4), organic acids (1), and amino acids (1) (Table 1). The characterization of the metabolites was carried out according to strategies published in several studies according to their retention time (RT min), observed masses (*m*/*z*, [M-H]^−^), molecular formulae, and UV absorption maxima [21,22,23,24,25]. The data were verified by comparing relevant literature and consulting several databases [7,26,27,28,29,30,31,32,33,34]. Moreover, Figure 2 portrays the structure of the major bioactive identified metabolites in the different parts of *R. vesicarius*.

#### 2.1.1. Phenolic Compounds

##### Phenolic Acids and Phenols

**Figure 3:**
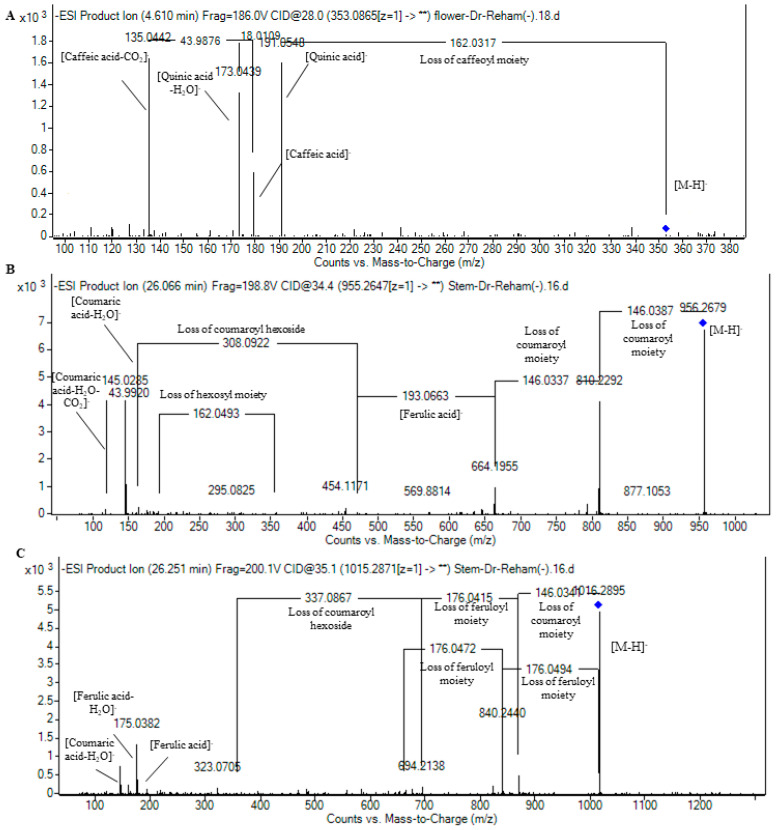
Pattern of fragmentation of (**A**) caffeoylquinic acid III, (**B**) vanicoside B I, and (**C**) lapathoside B I.

One hydroxybenzoic acid was annotated with *m*/*z* 153.019, expressing decarboxylation, and hence was characterized as dihydroxybenzoic acid [35]. As for hydroxycinnamic acids, they were distinctive markers in differentiating the studied parts, where three isomers of caffeoyl quinic acid were noticed only in the flowers and were characterized by the appearance of quinic acid at *m*/*z* 191.05, followed by its dehydrated ion at *m*/*z* 173.05, as well as the presence of the caffeic acid ion at *m*/*z* 179.03 followed by its dehydrated ion at *m*/*z* 161.02, and decarboxylated ion at *m*/*z* 135.04 [22]. In this line, Figure 3A demonstrates the fragmentation pattern of caffeoylquinic acid III. Furthermore, eight hydroxycinnamic acids were noticed only in the stems for the first time in the genus *Rumex*. They occurred as esters of coumaric acid and/or ferulic acid moieties with sucrose. They portrayed the loss of coumaroyl moieties at *m*/*z* 146.03, coumaric acid at *m*/*z* 163.04, feruloyl moieties at *m*/*z* 175.04, and/or ferulic acid at *m*/*z* 193.05. Their occurrence and fragmentation are in agreement with several reports of Polygonaceae [31,32,33,34]. In this context, helonioside B was noticed with *m*/*z* 735.21 expressing the neutral loss feruloyl and ferulic acid moieties as 6′-acetyl-3,6-diferuloylsucrose. Hydropiperoside (*m*/*z* 779.22) also portrayed the neutral loss of two coumaroyl moieties followed by dihexosyl moieties loss as β-d-(1,3,6-tri-*p*-coumaryl)-fructofuranosyl-α-d-glucopyranoside [22,32]. Two isomers of vanicoside B (*m*/*z* 955.27) were observed, showing the sequential loss of dicoumaroyl moieties followed by the neutral loss of ferulic acid and a coumaroyl hexosyl moiety as tricoumaroyl monoferuloyl sucrose, Figure 3B [34]. Similarly, two isomers of lapathoside A (*m*/*z* 985.28) expressed the sequential loss of coumaroyl, feruloyl, and hexosyl moieties as 1,6′-diferuloyl-3,6-di-*p*-coumaroyl sucrose. Furthermore, lapathoside B isomers I-II (*m*/*z* 1015.30) expressed a similar pattern of fragmentation to that of lapathoside A, with a loss of feruloyl moiety instead of a coumaroyl one, as 1,6,6′-triferuloyl-3-*p*-coumaroyl-sucrose. Figure 3C illustrates the fragmentation pattern of lapathoside A I.

Moreover, 6-gingerol and 8-gingerol were annotated in all parts of *R. vesicarius* except for the stem in the case of 8-gingerol. It is worth noting that their description is the first in *Rumex*, and their fragmentation pattern is in agreement with a previous report with the loss of the neutral alkyl moiety CH_3_(CH_2_)_4_CHO among other fragments [36].

**Table 1:** Metabolites characterized in the flowers, leaves, stems, and roots of *R. vesicarius* L.

Peak No.	RT (min)	[M − H]^−^	[M + H]^+^	(M)	Molecular Formula	Score	Error (ppm)	Main Fragments	DBE	Proposed Compound	Subclass	References	Peak Areas			
													Flowers	Leaves	Stems	Roots
1	0.69	387.1137		388.1209	C_13_H_24_O_13_	96.98	2.18	341.1073, 179.0566, 119.0342, 89.0244, 71.0139	2	Gluco-hepatonic acid hexoside	Su	[30]	1.05 × 10^4^	1.86 × 10^4^	3.78 × 10^3^	2.58 × 10^5^
2	0.76	341.1093		342.1162	C_12_H_22_O_11_	94.78	−0.8	179.0536	2	Disaccharide	Su	[14,25]	0	0	0	1.05 × 10^5^
3	0.82	431.1399		432.1742	C_15_H_28_O_14_	98.18	1.71	341.1071, 179.0549, 119.0343, 89.0241, 71.0137	2	Disaccharide glycerol I	Su	[29]	0	0	0	2.78 × 10^5^
4	0.90	191.0557		192.0634	C_7_H_12_O_6_	85.63	2.2	173.0473, 127.0347	2	Quinic acid	Oa	[25,35]	8.33 × 10^4^	0	0	0
5	0.94	431.1407		432.1742	C_15_H_28_O_14_	99.05	−0.3	341.1068, 179.0562, 119.0340, 89.0243, 71.0147	2	Disaccharide glycerol II	Su	[29]	0	0	0	4.42 × 10^5^
6	1.03		132.1013	131.0949	C_6_H_13_NO_2_	90.3	2.8	N.D.	1	Leucine/Isoleucine	Aa	[37,38]	8.61 × 10^4^	0	0	0
7	1.80	153.0186		154.0266	C_7_H_6_O_4_	82.92	4.54	152.0112, 125.0242, 124.0164, 109.0291, 108.0218, 107.0137	5	Dihydroxybenzoic acid	HB	[21,22]	1.09 × 10^5^	0	0	0
8	2.09	353.087		354.0951	C_16_H_18_O_9_	80.92	2.22	191.0529, 179.0324, 135.0442	8	Caffeoylquinic acid I	HC	[21,22]	5.03 × 10^4^	0	0	0
9	3.76	353.0866		354.0951	C_16_H_18_O_9_	89.66	3.33	191.0551, 173.0449, 161.0238	8	Caffeoylquinic acid II	HC	[21,22]	4.63 × 10^5^	0	0	0
10	4.56	353.0863		354.0951	C_16_H_18_O_9_	90.71	4.67	191.0543, 173.0449, 135.0446	8	Caffeoylquinic acid III	HC	[21,22]	1.92 × 10^5^	0	0	0
11	13.73	563.1391		564.1479	C_26_H_28_O_14_	94.73	2.18	545.1298, 503.1155, 473.1084, 443.0963, 413.0882, 383.0759, 353.0643	13	Apigenin-*C*-pentoside-C-hexoside I	Fla	[22,35]	5.00 × 10^4^	1.64 × 10^4^	0	0
12	14.06	447.0921	449.1047	448.1006	C_21_H_20_O_11_	96.55	2.67	357.0597, 327.0495, 297.0394, 151.0380, 133.0290	12	Luteolin-*C*-hexoside I	Fla	[21,22]	7.42 × 10^5^	0	3.82 × 10^5^	0
13	14.40	563.1391		564.1479	C_26_H_28_O_14_	93.05	2.22	545.1287, 503.1161, 473.1078, 443.0965, 413.0862, 383.0762, 353.0651, 117.0321	13	Apigenin-*C*-pentoside-C-hexoside II	Fla	[22,35]	2.29 × 10^5^	1.36 × 10^5^	0	0
14	14.40	447.0931	449.1062	448.1006	C_21_H_20_O_11_	96.81	1.10	357.0599, 327.0497, 297.0400	12	Luteolin-*C*-hexoside II	Fla	[21,22]	2.41 × 10^5^	0	1.41 × 10^5^	0
15	14.74	415.1235		416.1309	C_18_H_24_O_11_	79.95	2.61	207.0629, 193.0443, 192.0415	7	10-*O*-acetylgeniposidic acid	Tr	[30]	0	5.94 × 10^4^	0	0
16	15.15	563.1400		564.1479	C_26_H_28_O_14_	91.31	0.60	545.1366, 503.1144, 473.1106, 443.0963, 413.0834, 383.0775, 353.0653	13	Apigenin-*C*-pentoside-C-hexoside III	Fla	[22,35]	5.01 × 10^4^	2.10 × 10^4^	0	0
17	15.59	563.1391		564.1479	C_26_H_28_O_14_	92.70	2.84	545.1250, 503.1098, 473.1091, 443.0967, 413.0783, 383.0747, 353.0636, 117.0324	13	Apigenin-*C*-pentoside-C-hexoside IV	Fla	[22,35]	3.40 × 10^4^	1.61 × 10^4^	0	0
18	15.71	431.0978	433.1131	432.1056	C_21_H_20_O_10_	96.05	1.21	341.0636, 311.0557, 117.0319	12	Apigenin-*C*-hexoside I	Fla	[30]	9.58 × 10^5^	0	1.83 × 10^5^	0
19	15.90	431.0979	433.1124	432.1056	C_21_H_20_O_10_	98.57	1.42	341.0658, 311.0549, 117.0343	12	Apigenin-*C*-hexoside II	Fla	[30]	8.29 × 10^5^	0	5.14 × 10^5^	0
20	15.98	463.0868		464.0941	C_21_H_20_O_12_	84.12	1.98	301.0359, 300.0260, 271.0224, 255.0285, 178.9977, 151.0028	14	Quercetin-*O*-hexoside	Flo	[21,22,28]	9.23 × 10^4^	0	0	0
21	16.57	447.0931		448.1006	C_21_H_20_O_11_	96.81	1.10	285.0396	12	Luteolin-*O*-hexoside	Fla	[25,30]	9.70 × 10^4^	0	4.34 × 10^4^	0
22	17.23	187.0967		188.1049	C_9_H_16_O_4_	96.23	4.4	125.0966, 97.0656	2	Azelaic acid	Oa	[24,25]	5.20 × 10^4^	4.36 × 10^5^	0	0
23	17.49	607.1305		608.1366	C_27_H_28_O_16_	84.12	1.98	505.0970, 463.0873, 301.0336, 300.0264, 271.0257, 255.0277, 178.9982	14	Quercetin-*O*-hydroxymethylglutaryl-hexoside	Flo	[14]	8.73 × 10^4^	0	0	0
24	17.95	505.0966	529.0934 *	506.105	C_23_H_22_O_13_	71.34	3.58	463.0895, 301.0341, 300.0263, 271.0245, 255.0285, 178.9969, 151.0021	13	Quercetin-*O*-acetylhexoside I	Flo	[30]	3.27 × 10^4^	0	0	0
25	18.38	431.0973		432.1056	C_21_H_20_O_10_	81.61	2.36	269.0443, 268.0367	12	Apigenin-*O*-hexoside I	Fla	[22,30]	3.48 × 10^4^	3.85 × 10^4^	2.83 × 10^4^	0
26	19.02	649.1401		650.1467	C_29_H_30_O_17_	83.5	1.07	N.D.	15	**Quercetin-*O*-hydroxymethylglutaryl acetylhexoside I**	Flo		6.02 × 10^4^	0	0	0
27	19.08	505.0979		506.105	C_23_H_22_O_13_	97.66	1.95	463.0920, 301.0337, 300.0266, 271.0244, 255.0299, 151.0015	13	Quercetin-*O*-acetylhexoside II	Flo	[30]	3.70 × 10^5^	8.40 × 10^3^	0	0
28	19.59	431.0969		432.1056	C_21_H_20_O_10_	94.62	3.25	269.0441, 268.0369	12	Apigenin-*O*-hexoside II	Fla	[22,30]	1.36 × 10^5^	0	9.39 × 10^3^	0
29	20.19	649.139		650.1467	C_29_H_30_O_17_	85.47	2.53	587.1376, 505.0973, 463.0835, 301.0341, 300.0263, 271.0196, 255.0196, 178.9968, 151.0017	15	**Quercetin-*O*-hydroxymethylglutaryl acetylhexoside II**	Flo		6.93 × 10^4^	0	0	0
30	20.97	649.1389		650.1467	C_29_H_30_O_17_	86.02	3.81	587.1365, 505.0968, 463.0818, 301.0327, 300.0261, 271.0219, 255.0196, 178.9967	15	**Quercetin-*O*-hydroxymethylglutaryl acetylhexoside III**	Flo		7.53 × 10^4^	0	0	0
31	21.90	285.0392		286.0469	C_15_H_10_O_6_	79.66	4.0	151.0009, 133.0289	11	Luteolin	Fla	[21]	4.47 × 10^4^	0	4.66 × 10^4^	0
32	22.59	431.0972		432.1056	C_21_H_20_O_10_	94.74	−0.9	269.0441, 268.0369, 255.0620, 225.0554	12	Apigenin-*O*-hexoside III	Fla	[22,30]	3.63 × 10^5^	3.11 × 10^4^	2.21 × 10^5^	4.60 × 10^4^
33	22.61	735.2125		736.2198	C_39_H_40_O_19_	80.22	2.81	560.1761, 367.1231, 193.0506, 175.0382	15	Helonioside B	HC	[31]	0	0	4.00 × 10^4^	0
34	22.86	517.0997		518.1046	C_24_H_22_O_13_	82.33	0.17	N.D.	14	Quercetin-*O*-diacetylpentoside I	Flo	[39]	1.51 × 10^4^	0	0	0
35	23.42	389.1448	413.1406 *	390.1516	C_17_H_26_O_10_	82.94	1.28	227.0728, 189.0752, 145.0495, 127.0395, 83.0499	5	Loganin	Tr	[30]	7.80 × 10^4^	4.44 × 10^4^	1.46 × 10^5^	1.07 × 10^4^
36	23.51	517.0973		518.1046	C_24_H_22_O_13_	86.03	1.47	475.0975, 301.03327, 300.0261, 271.0201, 255.0266, 178.9967	14	Quercetin-*O*-diacetylpentoside II	Flo	[39]	1.05 × 10^5^	0	1.83 × 10^4^	1.72 × 10^3^
37	23.74	269.0442		270.0528	C_15_H_10_O_5_	96.2	4.6	225.0509, 151.0038, 117.0344	11	Apigenin	Fla	[22,28]	2.43 × 10^4^	0	3.59 × 10^4^	0
38	24.20	779.2185		780.2251	C_39_H_40_O_19_	95.07	1.44	634.1851, 633.1764, 488.1500, 487.1413, 163.0387, 145.0287	20	Hydropiperoside	HC	[32]	0	0	6.07 × 10^4^	0
39	24.31	285.0390		286.0469	C_15_H_10_O_6_	91.06	5.5	257.0450, 227.0341	11	Kaempferol	Flo	[22,28]	1.23 × 10^5^	0	0	0
40	25.75	187.1333		188.1405	C_10_H_20_O_3_	97.44	3.74	168.7586, 125.2039	1	Hydroxydecanoic acid	Fa	[30]	0	7.95 × 10^5^	0	0
41	26.07	955.2654		956.2717	C_49_H_48_O_20_	82.94	1.28	810.2292, 664.1955, 471.1293, 356.1108, 193.0473, 163.0371, 145.0285, 119.0451	26	Vanicoside B I	HC	[34]	0	0	1.85 × 10^5^	0
42	26.13	315.0502		316.0572	C_16_H_12_O_7_	90.09	3.37	300.0249, 271.0239, 255.0215	11	Isorhamnetin	Flo	[28,35]	3.94 × 10^4^	0	0	0
43	26.17	985.275		986.2825	C_50_H_50_O_21_	92.79	2.2	810.2335, 664.2372, 502.0949, 193.0443, 175.0392, 145.0303, 119.0451	26	Lapathoside A I	HC	[33]	0	0	1.18 × 10^5^	0
44	26.29	1015.289		1016.2925	C_51_H_52_O_22_	91.96	1.03	870.2553, 840.2440, 694.2138, 664.1926, 357.1271, 193.0485, 175.0382, 145.0274	26	Lapathoside B I	HC	[33]	0	0	9.33 × 10^4^	0
45	26.41	955.2657		956.2717	C_49_H_48_O_20_	79.61	2.47	810.2354, 664.2075, 163.0393, 145.0269, 119.0451	26	Vanicoside B II	HC	[34]	0	0	2.87 × 10^4^	0
46	26.41	985.2775		986.2825	C_50_H_50_O_21_	89.36	1.06	N.D.	26	Lapathoside A II	HC	[33]	0	0	2.66 × 10^4^	0
47	26.50	1015.285		1016.2925	C_51_H_52_O_22_	86.13	1.5	N.D.	26	Lapathoside B II	HC	[33]	0	0	2.87 × 10^4^	0
48	27.39	293.1748	295.1913	294.1818	C_17_H_26_O_4_	91.2	3.6	277.2879, 221.1539, 177.01912	5	6-Gingerol	Ph	[30,36]	2.01 × 10^5^	1.27 × 10^5^	2.55 × 10^5^	1.91 × 10^5^
49	27.59	213.1489		214.1562	C_12_H_22_O_3_	71.11	4.2	153.192	2	Hexanoic anhydride	Fa	[30]	0	0	0	5.33 × 10^4^
50	27.96		395.2197	394.2124	C_25_H_30_O_4_	99.8	−2.2	N.D.		Kazinol A	Hf	[30]	0	0	0	1.60 × 10^5^
51	28.46	269.0442		270.0528	C_15_H_10_O_5_	96.2	4.6	241.0492, 225.0544	11	Galangin	Flo	[30]	4.14 × 10^5^	1.33 × 10^4^	5.88 × 10^5^	4.92 × 10^5^
52	28.75	293.2105		294.2211	C_18_H_30_O_3_	90.5	5.2	275.2024, 231.2131	4	Hydroxylinolenic acid I	Fa	[30]	1.43 × 10^5^	8.59 × 10^5^	6.80 × 10^4^	0
53	29.26	295.2269		296.2364	C_18_H_32_O_3_	95.3	3.3	277.2158, 232.8021	3	Hydroxylinoleic acid I	Fa	[30,40]	6.40 × 10^5^	1.44 × 10^6^	4.09 × 10^5^	0
54	29.3		345.2048 *	322.2153	C_19_H_30_O_4_	98.8	2.2	279.2286, 179.0477	5	8-Gingerol	Ph	[30,36]	8.91 × 10^4^	7.02 × 10^4^	0	7.72 × 10^4^
55	29.37	297.2427		298.2520	C_18_H_34_O_3_	96.7	3.1	279.2303, 233.181	2	Hydroxyoleic acid I	Fa	[24,30]	0	1.37 × 10^5^	0	0
56	29.56	293.2118	317.2079 *	294.2211	C_18_H_30_O_3_	80.9	1.8	249.2155	4	Hydroxylinolenic acid II	Fa	[30]	2.13 × 10^5^	3.87 × 10^5^	1.52 × 10^5^	0
57	29.56	297.2427		298.2520	C_18_H_34_O_3_	97.6	2.8	279.2313	2	Hydroxyoleic acid II	Fa	[24,30]	1.10 × 10^5^	7.65 × 10^5^	5.82 × 10^4^	0
58	29.74	293.2118	317.2088 *	294.2211	C_18_H_30_O_3_	80.9	1.8	249.2094	4	Hydroxylinolenic acid III	Fa	[30]	0	0	2.29 × 10^5^	0
59	29.81	297.2425		298.2520	C_18_H_34_O_3_	90.6	4.3	279.2310, 233.1859	2	Hydroxyoleic acid III	Fa	[24,30]	5.04 × 10^5^	6.40 × 10^5^	2.33 × 10^5^	0
60	30.17	295.2266	319.2224 *	296.2364	C_18_H_32_O_3_	94.0	3.8	277.2160, 261.0815, 232.92, 199.1686	3	Hydroxylinoleic acid II	Fa	[30,40]	2.73 × 10^5^	1.48 × 10^5^	7.01 × 10^4^	0

* Ion with sodium adduct. Aa: amino acids, HC: hydroxycinnamic acids, HB, hydroxybenzoic acids, Hf: hydroxyflavonoid, Fa: fatty acids, Fla: flavones, Flo: flavonols, Oa: organic acids, Ph: phenol, Su: sugars, Tr: terpenes, N.D.: undetected, DBE: double-bond equivalence. Compounds in bold indicate new proposed structures. Peak area: lowest value 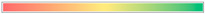 highest value.

#### 2.1.2. Flavonoids

With regards to flavonoids, they were a distinctive marker in differentiating flowers and stems from other parts in general; they were most abundant in flowers in qualitative and quantitative aspects (Figure 1A,E). From this perspective, they were classified into flavones (14 derivatives), flavonols (12), and a hydroxyflavonoid (Table 1).

##### Flavones

**Figure 4:**
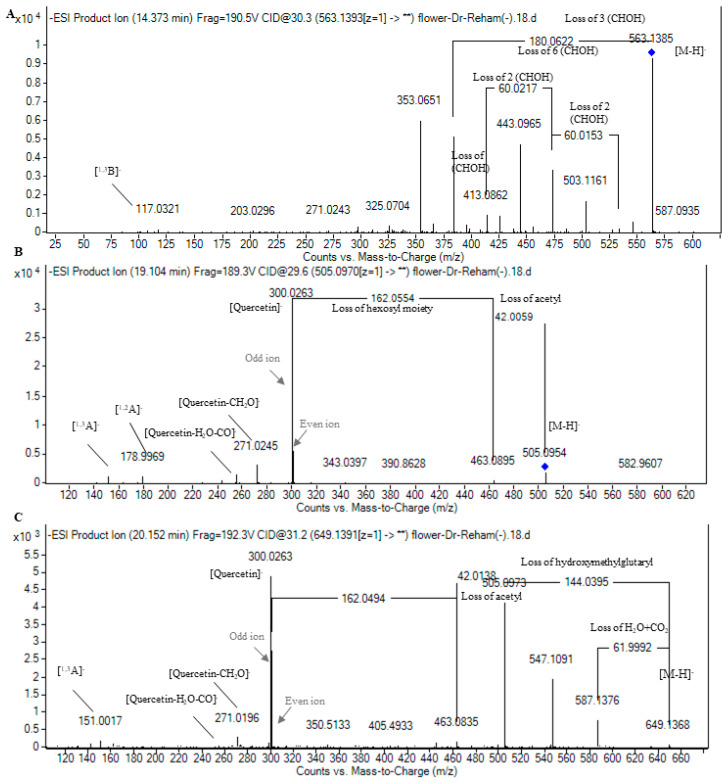
Pattern of fragmentation of (**A**) apigenin-*C*-pentoside hexoside II, (**B**) quercetin-*O*-acetylhexoside II, (**C**) quercetin-*O*-hydroxymethylglutaryl acetylhexoside II.

Flavones were derivatives of apigenin and luteolin. In this context, peak 37 with *m*/*z* 269.04 and molecular formula C_15_H_10_O_5_ indicated the presence of apigenin innate fragments of *m*/*z* 225.05 accounting for apigenin -CO_2_ and 151.00 and 117.03 accounting for the characteristic fragments of retro-Diels–Alder (RDA) reactions of flavonoids for the ions [^1,3^A]^−^ and [^1,3^B]^−^, respectively [22,41]. Moreover, five constitutional isomers were noticed for apigenin hexoside with *m*/*z* 431.10 and molecular formula C_21_H_20_O_10_. In fact, the employment of the state-of-the-art technique of RP-HPLC coupled with high-resolution QTOF-MS and tandem MS/MS enabled the differentiation between such constitutional isomers, where two of them occurred as apigenin-*C-*hexoside I-II expressing the fragmentation patten of *C*-glycosides with the neutral loss of n(CHOH, 30 Da) moieties [21,22], whereas three isomers were characterized as apigenin-*O*-hexoside expressing a hexosyl moiety loss (162 Da) and apigenin aglycone (*m*/*z* 269.04) [21,25]. It is worth mentioning that apigenin-*C-*hexoside was reported in *Rumex* as vitexin and isovitexin, while apigenin-*O*-hexoside was reported as apigenin-7-*O*-glucoside [30]. Also, four isomers with *m*/*z* 563.14 expressing the fragmentation pattern of *C*-glycosides with the appearance of the ions of *m*/*z* 117.03 for the [^1,3^B]^−^ characteristic apigenin ion were annotated as apigenin-*C*-pentoside-*C*-hexoside I-IV [35] and were described for the first time in *Rumex*, where it was observed in Polygonaceae as isoschaftoside [30]. Figure 4A demonstrates the pattern of fragmentation of apigenin-*C*-pentoside-*C*-hexoside II. Concerning luteolin derivatives, luteolin aglycone itself was detected with the characterized ions of retro-Diels–Alder (RDA) fragmentation of [^1,3^A]^−^ and [^1,3^B]^−^ of *m*/*z* 151.00 and 133.03, respectively [22,41]; see Table 1. Additionally, three constitutional isomers of luteolin hexoside were noticed where two isomers exhibited *C*-glycosylation fragmentation patterns, while the former one portrayed an *O*-glycosylation fragmentation pattern with the appearance of luteolin aglycone at *m*/*z* 285.04 [25,35]. It is noteworthy that orientin, isoorientin, and luteolin-7-*O*-glucoside were reported in *Rumex* [30].

##### Flavonols

Twelve flavonol derivatives were detected, mainly in the flowers of *R. vesicarius*, with the presence of galangin (*m*/*z* 269.04), kaempferol (*m*/*z* 285.04), and isorhamnetin (*m*/*z* 315.05) as aglycones (Table 1). Regarding quercetin derivatives, they occurred mainly as glycosides showing the neutral loss of conjugated moieties followed by the appearance of quercetin aglycone at *m*/*z* 301.03 with feature fragments of quercetin, *viz*., quercetin-CH_2_O (*m*/*z* 271.02), quercetin-H_2_O-CO (*m*/*z* 255.02), [^1,2^A]^−^ (*m*/*z* 179), and [^1,3^A]^−^ (*m*/*z* 151) [22,41]. In this regard, peak 20 was annotated as quercetin-*O*-hexoside with a hexosyl moiety loss (162 Da) alongside quercetin fragmentation. It bears noting that it was described in *R. aquaticus* as quercetin-3-*O*-β-D-galactopyranoside [28]. Peaks 24 and 27 exhibited the same fragmentation pattern as peak 20 with an additional loss of an acetyl moiety (42 Da) and hence were characterized as quercetin-*O*-acetylhexoside I and II (Figure 4B) and described in Polygonaceae as quercetin-3-*O*-(6″-acetyl-glucoside). Likewise, two isomers of quercetin-*O*-diacetylpentoside indicated the neutral loss of two acetyl and one pentosyl moieties followed by quercetin fragmentation (Table 1). Peak 23 showed a quercetin-*O*-hexoside fragmentation pattern with a prior loss of a hydroxymethylglutaryl moiety (*m*/*z* 144 Da) and hence was characterized as quercetin-*O*-hydroxymethylglutaryl hexoside, which was observed in *R. tunetanus* [14]. Furthermore, three peaks were observed with a fragmentation pattern similar to that of the aforementioned compound with an additional loss of an acetyl moiety (42 Da) (Table 1), and they were characterized as quercetin-*O*-hydroxymethylglutaryl acetylhexoside I-III as new proposed structures (Figure 4C).

A hydroxyflavonoid was detected in the roots with *m*/*z* (395.22) and molecular formula C_25_H_30_O_4_ and was characterized as kazinol A (Table 1) [30].

#### 2.1.3. Terpenes

Loganin (C_17_H_26_O_10_, *m*/*z* 389.15) was observed in all the parts of the *R. vesicarius* extracts as examples of sesquiterpene, whereas 10-*O*-acetylgeniposidic acid (C_18_H_24_O_11_, *m*/*z* 415.12) was noticed only on the leaves as an example of iridoid glycosides (Table 1) [30].

#### 2.1.4. Fatty Acids

Ten fatty acid derivatives were noticed and were classified into short-chain fatty acids as hydroxydecanoic acid and hexanoic acid anhydride, where the former was observed only in the leaves while the latter was noticed only in the roots (Table 1) [30].

As for long-chain fatty acids, three isomers of hydroxylinolenic acid, two isomers of hydroxylinoleic acid, and three isomers of hydroxyoleic acid were characterized in different parts of *R. vesicarius* [24,40,42].

#### 2.1.5. Amino Acids, Organic Acids, and Sugars

Leucine/isoleucine and quinic acids were noticed in the flowers only, and azelaic acid was present in both the flowers and leaves (Table 1) in agreement with previous reports [22,35].

Four sugar derivatives were found only in the roots, namely a disaccharide, two isomers of disaccharide glycerol, and gluco-hepatonic acid hexoside [14,30].

### 2.2. Comparison of the R. vesicarius Different Parts by Multivariate Data Analysis

**Figure 5:**
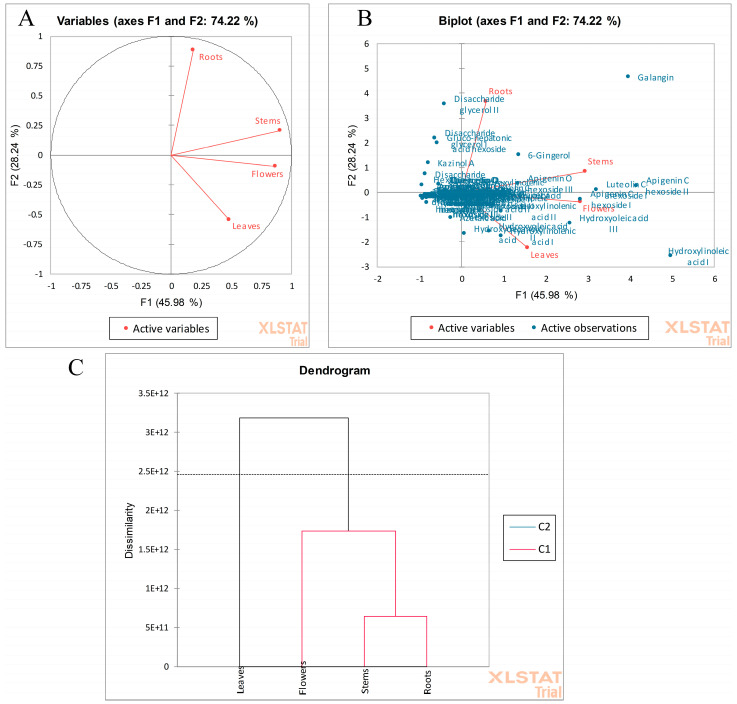
(**A**) PCA score plot and (**B**) biplot. (**C**) HCA dendogram based on RP-HPLC-QTOF-MS/MS analysis of *R. vesicarius* flowers, leaves, stems, and roots.

Multivariate data analysis was employed, considering the relative abundance of the characterized metabolite peaks, for discrimination between the different parts in the conducted study by principal components analysis (PCA) and hierarchical clustering analysis (HCA) [22,43]. In this sense, the first three components PCA demonstrated made up 93.57% of the total variance in the score plot. Both the roots and the stems were in one quadrant, as were the flowers and leaves, yet the flowers and stems were closely related to each other (Figure 5A). The biplot of variables and observations showed several metabolites that contributed to the parts’ segregation from each other, where some markers were distinguished and aided in parts segregation as galangin, kazinol A, 6-gingerol, vanicoside B I-II, lapathoside B I-II apigenin *C* hexoside I-II, disaccharide glycerol II, hydroxyoleic acid III, and hydroxylinoleic acid I (Figure 5B).

In the same manner, HCA revealed two main clusters where the leaves were distant from the other parts, as they were clustered in one main cluster while the other cluster was for the remaining parts. Moreover, stems and roots were near each other, as they were located in a subcluster (Figure 5C). In this line, PCA and HCA served in the chemotyping of different *R. vesicarius* parts for marker distinction and/or to facilitate bioactive metabolites standardization of each part.

### 2.3. Antioxidant Evaluation of Different Parts of Rumex vesicarius Extracts

Oxidative stress is a state that occurs when there is an imbalance between the production of reactive oxygen species (ROS) and the body*’*s ability to neutralize them with antioxidants. ROS are highly reactive molecules that can damage lipids, proteins, and DNA if left unchecked [44].

Oxidative stress has been implicated in the pathogenesis of many diseases, including cancer, cardiovascular disease, neurodegenerative disorders, diabetes, and aging [45]. Overall, maintaining a healthy balance between ROS production and antioxidant defenses is critical for preserving cellular function and preventing oxidative damage [46]. Many natural plants have been investigated in cell culture and animal studies, as well as in some human clinical trials, for their potential to prevent or treat conditions linked to oxidative stress. The research is still emerging, but the evidence suggests natural antioxidants may be a useful complement to conventional therapies [47].

**Figure 6:**
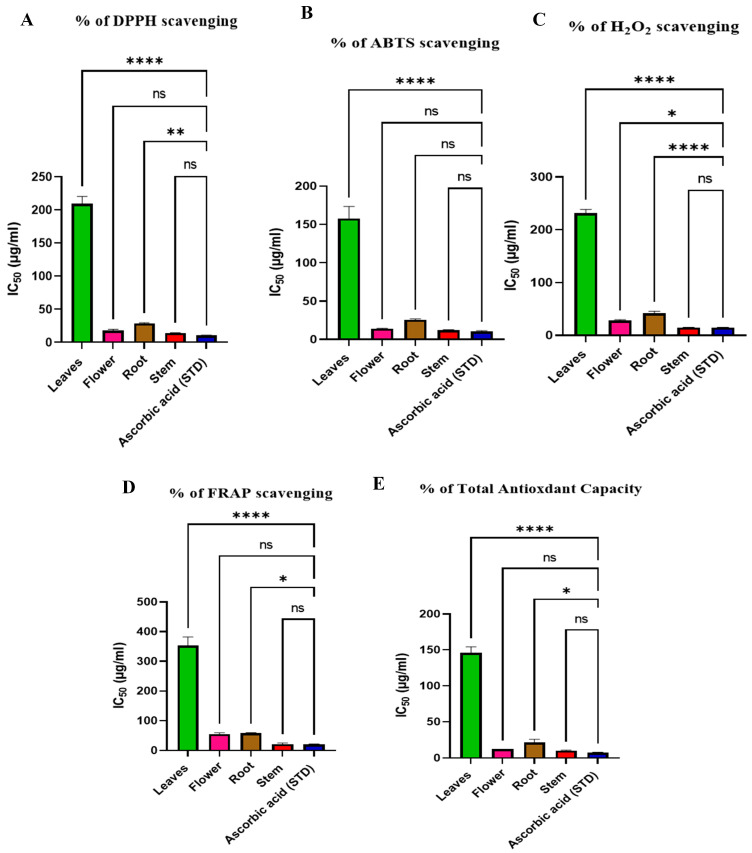
(**A**) DPPH, (**B**) ABTS, (**C**) H_2_O_2_, (**D**) FRAP, and (**E**) TAC scavenging activities for leaf, flower, root, and stem extracts and ascorbic acid as standard. Results are revealed as means ± S.D. (measured in triplicate; n = 3) using ANOVA followed by Tukey as a post-hoc test. ns: non-significant, **** means significant (*p* < 0.0001), ** means significant (*p* < 0.01), * means significant (*p* < 0.05).

Antioxidant activities are evaluated by measuring DPPH, ABTS, H_2_O_2_, FRAP, and TAC scavenging activities in leaf, flower, root, and stem extracts compared to ascorbic acid standard and to each other, as shown in Table S1 and Figure 6, and the determination of IC_50_ depends on the percentage of inhibition of activity, as shown in Tables S2–S6 and Figures S1–S5.

The results showed that the stems are the most active part, as they exhibited the highest antioxidant activity with IC_50_ 14.11 ± 0.52, 12.28 ± 0.47, 14.99 ± 0.11, 21.37 ± 4.01, and 10.16 ± 0.77 for DPPH, ABTS, H_2_O_2_, FRAP, and TAC scavenging activities, respectively, close to standard values, followed by flowers extract, which showed similar antioxidant values that were close to those of stems.

Our results agreed with previous works which suggest that *R. vesicarius* is rich in polyphenolic compounds, such as flavonoids, phenolic acids, and tannins [15]. These phytochemicals are known to have potent antioxidant and free radical scavenging abilities [48]. Several studies have shown that extracts of *R. vesicarius* exhibit strong free radical scavenging activity and reducing power and metal chelating ability in cell-free assays. The antioxidant capacity of the plant has been attributed to its high content of phenolic and flavonoid compounds [20].

*R. vesicarius* extracts have been found to enhance the activities of endogenous antioxidant enzymes, such as superoxide dismutase (SOD), catalase, and glutathione peroxidase, in experimental models [49]. In a previous study, *R. vesicarius* extracts demonstrated the ability to protect against oxidative stress-induced damage in various organs, including the liver, kidneys, and brain [50]. The antioxidant properties of the plant may contribute to its observed protective effects against conditions like diabetes, inflammation, and heavy metal toxicity [45]. Based on the available evidence, *R.vesicarius* shows promise as a natural source of antioxidants that could be useful in the prevention or management of diseases linked to oxidative stress. However, more clinical research is needed to fully understand the therapeutic potential of this plant and its active constituents. In summary, the robust antioxidant potential of *R. vesicarius* is primarily attributed to its rich phytochemical profile, particularly its high content of polyphenolic compounds. Further investigation is warranted to explore the possible therapeutic applications of this natural antioxidant source.

### 2.4. Docking against Antioxidant Molecular Targets

**Figure 7:**
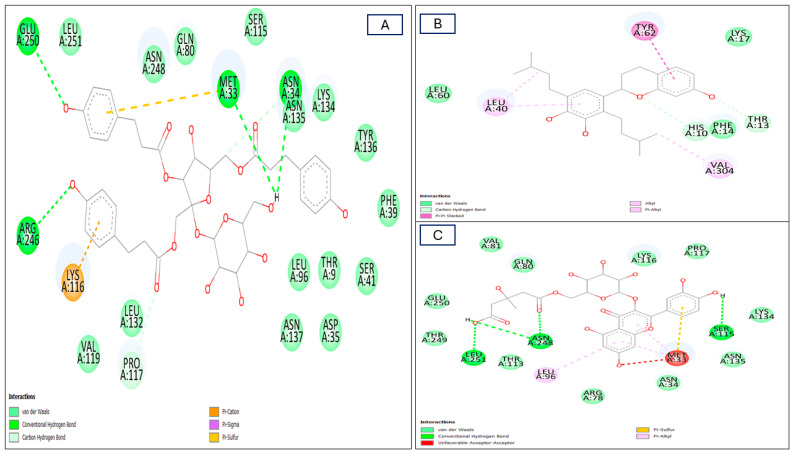
Two-dimensional (2D) molecular interactions of the most dominant chemical compounds: (**A**) compound **3**; (**B**) compound **6**; and (**C**) compound **10** identified in different *R. vesicarius* organs with the active site of the NADPH oxidase (NO) receptor (PDB ID:2CDU), (dimensions X: 19.1672, Y: 17.5909, Z: 29.7368), (root mean square deviation) RMSD < 2.

The virtual molecular study of the predominant polyphenol components, identified from various parts of *R. vesicarius* methanolic extracts, were carried out within the binding sites of NADPH oxidase (NO) and human peroxiredoxin 5 enzyme (PRDX5). The results are recorded in Table 2 and Figure 7 and Figure S6 and revealed that most of the examined compounds showed inhibitive activity against both tested enzymes, with varieties in the scores that showed similarity to FAD and benzoic acid co-crystallized ligands.

#### 2.4.1. Interactions with the NADPH Receptor

NADPH oxidase (NOX) is one of the crucial oxidative bursts and consists of seven members implicated in various vital human physiological processes that are medicated to release reactive oxygen species (ROS). ROS have two distinct responsibilities that change based on the situation. Numerous illnesses and dysfunctions, including immunodeficiency and lung and cardiovascular disorders have been linked to ROS-mediated stress. On the other hand, the NADPH oxidase (NOX)-mediated release of ROS results in the removal of invasive germs in neutrophils and macrophages and hence acts as an inflammatory mediator [51,52,53]. Kazinol A and quercetin-3-*O*-6″-(3-hydroxyl-3-methylglutaryl)-D-glucopyranoside, followed by hydropiperoside, exhibited the best fit position within the enzyme active site, more than the co-crystalline ligand, displaying binding affinities (BA) of −7.7, −7.6, and −7.5 Kcal/mol, respectively (Table 2). The formation of many linkages was explained by this fitting within the active site of the NADPH–oxidase enzyme. Hydropiperoside established four conventional-H bonds with Arg246, Asn34, Glu250, and Met33 and two C-H bonds with Asn34 and Pro117, together with three different pi-bonds including pi-cation and pi-sigma bonds with Lys116 and pi-sulphur bonds with Met33 together with the formation of multiple Van interactions (Figure 7A and Figure S6(3A)). Meanwhile, kazinol A established four hydrophobic bonds with Leu40, Tyr62, and Val304; two C-H bonds with His10 and Thr13; and many Van der Waals interactions (Figure 7B and Figure S6(6A)). Quercetin-3-*O*-6″-(3-hydroxyl-3-methylglutaryl)-D-glucopyranoside formed four conventional-H bonds with Asn248, Leu251, and Ser115 residues; three alkyl bonds with Leu96, and Met33; one pi-sulphur bond with Met33, together with many Van der Waals interactions (Figure 7C and Figure S6(10A)).

#### 2.4.2. Interactions with the Human Peroxiredoxin 5 Protein (PRDX5)

**Figure 8:**
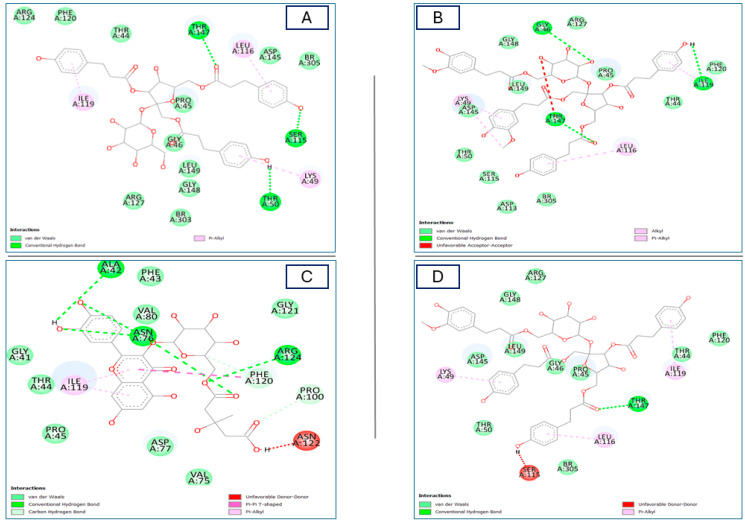
Two-dimensional (2D) molecular interactions of the most dominant chemical components: (**A**) compound **3**; (**B**) compound **7**; (**C**) compound **10**; and (**D**) compound **11** identified in different *R. vesicarius* organs with the active site of human peroxiredoxin 5 enzymes (PDB ID:1HD2), (dimensions X:22.5007, Y: 25.3708, Z:20.1494), (root mean square deviation) RMSD < 2.

A distinct redox-sensitive protein called human peroxiredoxin-5 (PRDX5) is involved in brain injury. While the functions of the intracellular PRDX5 have been reported as an antioxidative enzyme by scavenging peroxides, which is also considered a neuroprotective agent [54]. Concerning PRDX5, the tested compounds formed higher binding affinities rather than the benzoic acid (−4.0 Kcal/mol) as a co-crystallized ligand, and they showed higher binding affinities rather than FAD ligand (−6.1 Kcal/mol) except for four *C*-glycoside flavonoid derivatives and gentiobiosylglycerol, which explains the antioxidant activity of the *R. vesicarius* methanolic extracts of the two organs, including roots and stems. The binding affinities of the stable products range from −4.6 to −6.9 Kcal/mol (Table 2). Regarding the molecular docking results, quercetin-3-*O*-6″-(3-hydroxyl-3-methylglutaryl)-D-glucopyranoside showed the highest binding affinity (−6.9 Kcal/mol), followed by the hydroxy-phenolic derivatives hydropiperoside, lapathoside A, vanicoside B, lapathoside B, kazinol A, and helonioside B with −6.8, −6.4, −6.2, −6.1, −6.1, and −6.0 Kcal/mol binding affinities, respectively. Hydropiperoside formed three conventional H bonds with Ser115, Thr50, and Thr147; and three pi-alkyl bonds with Ile119, Leu116, and Lys49 (Figure 8A and Figure S6(3B)). Meanwhile, lapathoside A established six hydrophobic bonds, three of which were with Gly46, Ile119, and Thr147 as C-H bonds and with Ile119, Leu116, and Lys49 as alkyl bonds (Figure 8B and Figure S6(7B)). Quercetin-3-*O*-6″-(3-hydroxyl-3-methylglutaryl)-D-glucopyranoside formed five strong conventional H bonds with Ala42, Arg124, Asn76; two C-H bonds with Phe120 and Pro100; as well as a pi-pi T-shaped bond with Phe120; and pi-alkyl bond with Ile119 (Figure 8C and Figure S6(10B)). Vanicoside B formed three hydrophobic alkyl bonds with Ile119, Leu116, and Lys49 and one conventional H-bond with Thr147 (Figure 8D and Figure S6(11B)). All abovementioned compounds showed several Van der Waals interactions in Figure 8 and Figure S6B.

Regarding the in silico results for both enzymes, they supported the obtained in vitro results, wherein hydropiperoside, vanicoside B, lapathoside A and lapathoside B existed only in the stems and, for instance, kazinol A existed only in the roots when compared to other organs and it appears that the highest potent antioxidant activity is dependent on the subclass hydroxy-cinnamic acids present in the stems organ, as revealed in all the investigated assays.

### 2.5. ADME Predictions of the Selected Compounds

ADME parameters were estimated to the major compounds identified in the four different organs of *R. vesicarius* methanolic crude extract which make them a promising option for drug development. Interestingly, one phytoconstituent was found to achieve Lipinski’s rule of five with a good to poor bioavailability score of most phytoconstituents. It is also important to note that the majority of the compounds are shown to be soluble in water in the results that is a crucial property for absorption and distribution within the body. The possibility for developing a transdermal medicine delivery method must be evaluated by measuring the skin’s permeability, or the rate at which a molecule penetrates the stratum corneum. It was discovered that the crude extracts identified phytochemical ingredients, all had good skin penetrability. Finally, the majority of the detected phyto-compounds were discovered to be P-gp inhibitors or substrates. P-gp is the primary component of ATP-binding cassette transporters, also known as ABC transporters, which are employed to safeguard the central nervous system (CNS) against exogenous agents and are a primary technique for identifying active efflux across biological membranes. It was discovered that the majority of the phytoconstituents were not CYP3A4 inhibitors, which is the isoenzyme in charge of 60% of xenobiotic metabolism, which includes carcinogens, drugs, eicosanoids, and steroids.

ADME tool results showed that all compounds have poor bioavailability (0.11–0.17) except compounds **5**, **6**, and **12** with a high score (0.55), and only compound 6 fulfilled all drug-likeness rules without any violations. These three compounds had moderate synthetic accessibility (4.34–5.12), which showed an explicit synthetic route, meanwhile, other compounds (1–4, and 7–11) showed a moderate to difficult synthetic accessibility range from 5.04–8.12. In addition, all compounds were varied from being poor to being highly soluble in water, especially compound 1 was observed as a high soluble in water, on the other hand compounds **2**, **4**, **5**, **9**, **10**, and **12** were observed with good solubility and only compound 3 was showed as a moderately soluble in water.

**Figure 9:**
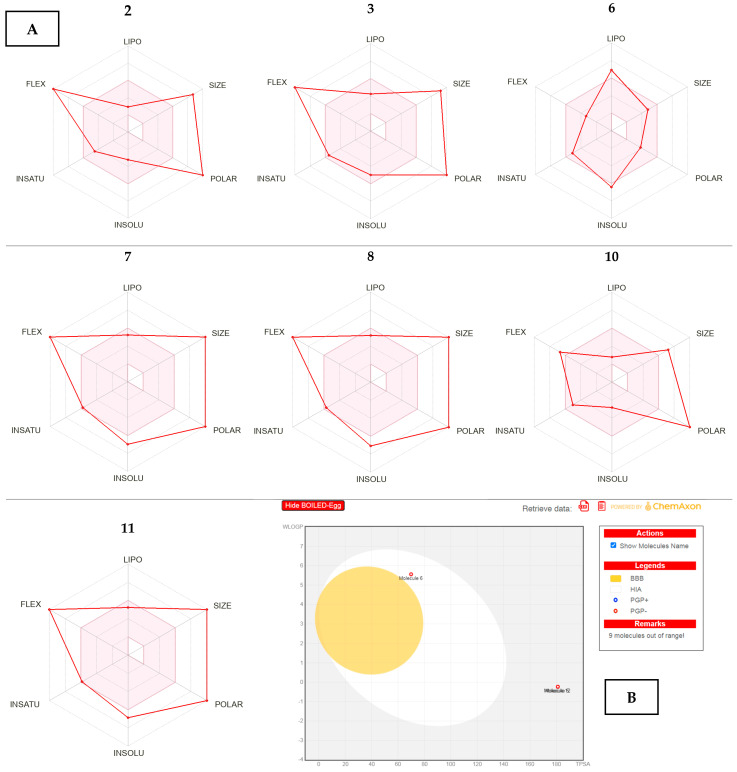
(**A**): Bioavailability diagram of selected molecules (2,3, 6–8, 10, and 11), (**B**): BOILED-Egg diagram.

Sweilam et al., 2023 with their co-workers mentioned that the absorption and diffusion of the compounds through the blood–brain barrier were predicted by using the BOILED-Egg tool [52]. It is worth mentioning that compound 6 was the only one that has high GIT absorption properties, on the other hand, all compounds (**1**–**12**) predicted poor diffusion through the BBB, and all have high permeability values for skin permeable parameters. Compound 1 is thought to enter the skin at a log Kp value of greater than 2.5 cm/h, which had the best skin penetrability, followed by compound 2 then compound 10. Seven compounds (**1**–**3**, **7**–**8**, and **10**–**11**) showed binding affinity to the P-gp substrate, whereas only compound 6 showed inhibition activity against CYP3A4 and CYP2C9 human isoforms. Skin permeation (log Kp), distribution, and ADME properties were analysed by the Swiss-ADME software and recorded in Table 3 and Figure 9. The final conclusions +revealed that one of the twelve compounds fulfilled the oral drug ability of Lipinski’s rule of five, with five slightly meeting the criteria of this rule.

## 3. Materials and Methods

### 3.1. Plant Material

#### 3.1.1. Plant Collection

The stems, flowers, leaves, and roots of (*Rumex vesicarius* L. family (Polygonaceae) were collected from Al-Sharqia Governorate, 30°15′49.9″ N 31°45′07.4″ E, Egypt during March 2020 (identified by Prof. Dr. Mohamed Elgebaly, Professor of Taxonomy, National Research Center, Giza, Egypt). Voucher specimens NO. ERU RV_20 is kept at the Department of Pharmacognosy, Faculty of Pharmacy, Egyptian Russian University Herbarium.

#### 3.1.2. Preparation of Different Parts Extracts

The dried powdered flowers, stems, leaves, and roots (200, 500, 100, and 10 g, respectively) were defatted with *n*-hexane followed by maceration in 80% methanol, (20 mL solvent for each gram of powder under ultrasonication for one hour per day twice) till exhaustion. Extracts were collected, filtered, pooled, and evaporated under vacuum (at 40 °C) until free from solvent to give dark residues (10.23, 24.23, 4.32, and 1.1 g) for the flowers, stems, leaves, and roots, respectively. All extracts were saved for further examination at −20 °C [23].

### 3.2. RP-HPLC-ESI-MS and Tandem MS/MS Analysis

Chromatographic separations were conducted using an Agilent 1200 series rapid resolution system (Agilent Technologies, Santa Clara, CA, USA), which included a quaternary pump (G7104C) and an autosampler (G7129A) using a Poroshell 120 HiLiC Plus column (150 mm × 3 mm, 2.7 μm particle size, Agilent Technologies) [24,25]. Acidified water (0.5% acetic acid, *v*/*v*) (phase A) and acetonitrile (phase B) were the mobile phases for gradient elution with a flow rate of 0.2 mL/min. The injection volume was 5 µL and the replicates of extracts were analyzed. The gradient elution was as follows: 0 minutes (99% A, 1% B), 5.50 minutes (93% A, 7% B), 11 minutes (86% A, 14% B), 17.50 minutes (76% A, 24% B), 22.50 minutes (60% A, 40 B), 27.50 minutes (0% A, 100% B), 28.50 minutes (0% A, 100% B), 29.50 minutes (99% A, 1% B), 35 minutes (99% A, 1% B).

The system was connected to a 6530-quadruple time of flight (Q-TOF) LC/MS (Agilent Technologies) that had a dual ESI interface, as described in references [22,25]. Regarding the operating conditions, they were governed by MassHunter Workstation software B.06.00 (Agilent technologies). Briefly, the drying gas temperature was 325 °C (10 L/min flow; 20 psig nebulizer pressure). The temperature of the sheath gas was 400 °C, and it flowed at a rate of 12 L/min. The capillary voltage was set at 4000 V, the nozzle voltage at 500 V, the fragmentor voltage at 130 V, the skimmer voltage at 45 V, and the octupole radiofrequency voltage at 750 V.The mass to charge (*m*/*z*) ranged from 70 to 1500 Da.

The data processing and characterization of metabolites were conducted using MassHunter Qualitative Processing B.06.00 (Agilent Technologies) as stated in references [21,24,25] taking into consideration observed *m*/*z*, RT, associated fragments for *m*/*z* and generated molecular formulae.

### 3.3. Determination of the Antioxidant Activity

#### 3.3.1. Evaluation of the Antioxidant Activity Using DPPH Scavenging

The 2,2-diphenyl-1-picrylhydrazyl (DPPH) radical was produced in a fresh methanol solution and kept in the dark at 10 °C. the test compound was prepared. A 40 μL aliquot of methanol was added to 3 mL of DPPH solution. Using a UV–visible spectrophotometer, absorbance values were promptly recorded (Milton Roy, Spectronic 1201). The absorbance for (control) and the reference compound ascorbic acid were also detected. All the determinations were performed in three times and averaged [56,57]. The percentage inhibition (PI) of the DPPH radical was calculated using the Formula (1):

PI = [(A_C_ − A_T_)/A_C_ × 100] (1)

where A_C_ = Absorbance of the control and A_T_ = absorbance of the sample + DPPH. The 50% inhibitory concentration (IC_50_), (the concentration needed to achieve 50% DPPH radical scavenging activity) was calculated from dose response curve graphic plots.

#### 3.3.2. Evaluation of the Antioxidant Activity Using ABTS Radical Scavenging

ABTS scavenging capacity is a decolorization assay which is determined according to the procedure described by others [57,58]. Measurements were taken at room temperature. The extracts were diluted at a ratio of 1:10 with (80%) methanol.

The absorbance at 734 nm was measured every minute for 13 min following initial mixing. Ascorbic acid and methanol were used as the standard antioxidant and the negative control, respectively. Experiments were performed three times with three replicates for each sample. The percent free radical scavenging activity was calculated according to Formula (2):

% Free Radical Scavenging Activity = [(A_n_ − A_s_) × 100]/A_n_ (2)

where A_n_ is the final absorbance values of the negative control, and A_s_ is the final absorbance values of the sample.

The 50% inhibitory concentration (IC_50_) was determined as showed in DPPH method.

#### 3.3.3. Evaluation of the Antioxidant Activity Using H_2_O_2_ Scavenging Assay

Antioxidant activity using H_2_O_2_ acavenging assay was performed as previously described [59]. The H_2_O_2_ radical scavenging percentage of the extracts was calculated using the following Formula (3):

H_2_O_2_ radical scavenging percentage = [(A_blank_ − A_sample_)/A_blank_] × 100 g extract. (3)

The 50% inhibitory concentration (IC_50_) was determined from graphic plots of the dose response.

#### 3.3.4. Evaluation of the Antioxidant Activity Using FRAP Scavenging

The reduction of ferric to ferrous ions by the extract is an indication of the potential antioxidant property. The reducing power of the extract was evaluated as previously described [60,61]. This method is based on the reduction of ferricyanide relative to extracts. Samples (1 mg/mL) in 1 mL of methanol were mixed with 2.5 mL of 0.2 M sodium phosphate buffer (pH 6.6) and 2.5 mL of potassium ferricyanide [K_3_Fe (CN)_6_] (1%, *w*/*v*). After 20 minutes of incubation at 50 °C, the reaction mixture was acidified with 2.5 mL of trichloroacetic acid (10%, *w*/*v*). The reaction mixture was centrifuged at 1000× *g* for 10 minutes. The supernatant solution (2.5 mL) was mixed with 2.5 mL of deionized water and 0.5 mL of freshly prepared ferric chloride (0.1%, *w*/*v*). The absorbance of the resulting solution was measured at 700 nm versus a blank using the previously mentioned spectrophotometer. The reducing capability percentage (%) was calculated as follows (4):

Reducing capability % = 100 − (A_0_ − A_s_/A_0_ × 100) (4)

where, A_0_: absorbance of the control solution. A_s_: sample absorbance.

The 50% inhibitory concentration (IC_50_) was calculated using the DPPH method.

#### 3.3.5. Determination of the Total Antioxidant Capacity (TAC)

The TAC was investigated by means of the method previously reported [62].

Ascorbic acid and methanol were used as the standard antioxidant and the negative control, respectively. Experiments were performed three times with three replicates for each sample. The total antioxidant activity percent was calculated according to the following Formula (5):

% Activity = [(A_n_ − A_s_) × 100]/An (5)

where A_n_ is the final absorbance values of negative control, and A_s_ is the final absorbance values of sample.

The 50% inhibitory concentration (IC_50_), was estimated from graphic plots of the dose response.

### 3.4. Statistical Analysis

Statistical analysis was done employing one-way analysis of variance (ANOVA) where the results are expressed as the mean ± standard deviation of the mean (SD) followed by Tukey as a post-hoc test. Values with *p* < 0.05 are considered significantly different using Graphpad Prism software 5.04 (San Diego, CA, USA), the conditional formatting was performed by Microsoft Excel 365 (Redmond, WA, USA), and the bubble plot was created by Minitab 17 (Minitab, Inc., State College, PA, USA) [21].

### 3.5. In Silico Docking Study and ADME Analysis

Docking analysis was performed for twelve predominant hydroxy-cinnamic acid and flavonoid derivatives (gentiobiosylglycerol, helonioside B, hydropiperoside, isoorientin, isovitexin, kazinol A, lapathoside A, lapathoside B, orientin, quercetin-3-*O*-6″-(3-hydroxyl-3-methylglutaryl)-β-D-glucopyranoside, vanicoside B, and vitexin), which were phytochemically identified from four different organs (roots, flowers, stems, and/or leaves) of *R. vesicarius*, two explored antioxidant targets, including NADPH oxidase (NO) (PDB ID: 2CDU; 1.80Å) receptor [63,64] and human peroxiredoxin 5 enzyme (PDB code: 1HD2, 1.50Å) [65,66] were downloaded from PDB (protein data bank, https://www.rcsb.org (accessed on 15 May 2024). The docking study was executed using PyRx Autodock Vina (Scripps Research, La Jolla, CA, USA) as previously published [42,52]. The hydroxy-cinnamic acid derivatives were predicted to show high affinities to the binding sites. Additionally, these compounds were examined for drug likeness, pharmacokinetics, physicochemical properties, and solubility by using the online free SwissADME site (http://www.swissadme.ch/, accessed on 22 May 2024).

## 4. Conclusions

The conducted study portrays a comprehensive metabolic profiling of different parts of *R. vesicarius*, revealing 60 metabolites. The stems were most abundant in hydroxycinnamic acids, whereas the flowers were most abundant in flavonoids. PCA and HCA enabled the discrimination of the studied parts. The stems showed antioxidant potential similar to ascorbic acid, the used standard. Overall, high concentrations of the polyhydroxy derivatives identified in the different organs of *R. vesicarius* ascertain its antioxidant characteristic properties. Further in vitro and in silico studies are needed to confirm this activity. Herein, future prospective in vivo pharmacological analysis is necessary to support the confirmation of these properties of the identified molecules as antioxidant agents.

## Figures and Tables

**Table 2:** Docking analysis of the predominant compounds identified in different *R. vesicarius* organs against antioxidant enzymes (binding energies, interaction type derived from the best conformations of each compound into the macromolecule).

No	Components	2CDU (BA)	No. of Formed Bonds/AA-Residues	1HD2 (BA)	No. of Formed Bonds/AA-Residues
1	Gentiobiosylglycerol	−6.3	2 */Asn34, Asn135, Asn248, Gln80, Glu250, Leu251, Lys116, Lys134, Met33, Pro117, Ser115, Thr113, Thr249	−4.6	2/Arg127, Gly46, Pro45, Thr147
2	Helonioside B	−7.0	5 */Asn34, Asn36, Asn134, Asn135, Asn137, Asn248, Asp138, Gln80, Glu32, Ile37, Leu132, Leu251, Lys116, Met33, Pro117, Ser41, Thr9, Thr82, Thr113, Tyr136, Val81, Val119	−6.0	3/Arg124, Arg127, Asp145, Br303, Gly46, Gly148, Ile119, Leu116, Leu149, Phe43, Phe120, Pro45, Thr44, Thr147
3	Hydropiperoside	−7.5	6/Arg246, Asn34, Asn135, Asn137, Asn248, Asp35, Gln80, Glu250, Leu96, Leu132, Leu251, Lys116, Lys134, Met33, Phe39, Pro117, Ser41, Ser115, Thr9, Tyr136, Val119	−6.8	3/Arg124, Arg127, Asp145, Br303, Br305, Gly46, Gly148, Ile119, Leu116, Leu149, Lys49, Phe120, Pro45, Ser115, Thr44, Thr50, Thr147
4	Isoorientin	−6.8	3 */Asn34, Asn36, Asn135, Asn248, Ile37, Lys116, Lys134, Met33, Phe39, Pro117, Ser41, Thr9, Tyr136	−6.0	4/Ala42, Arg124, Asn76, Gly41, Ile119, Phe43, Phe120, Pro45, Thr44, Val80
5	Isovitexin	−6.5	3 */Asn34, Asn135, Asn248, Leu132, Lys116, Lys134, Met33, Phe39, Pro117, Ser115, Thr113, Tyr136	−5.9	4/Arg124, Gly41, Ile119, Phe43, Phe120, Pro45, Thr44, Val80
6	Kazinol A	−7.7	5/His10, Leu40, Leu60, Lys17, Phe14, Thr13, Tyr62, Val304	−6.1	5/Arg127, Asp145, Br305, Gly46, Gly148, Leu112, Leu116, Leu149, Ile119, Pro45, Ser115, Thr147
7	Lapathoside A	−6.7	6/Arg78, Asn34, Asn135, Asn137, Asn248, Asp35, Asp138, Gln80, Glu250, Leu96, Leu132, Lys116, Lys134, Met33, Phe39, Pro117, Ser41, Ser115, Thr9, Tyr136, Val119	−6.4	4 */Arg127, Asp113, Asp145, Br305, Gly46, Gly148, Ile119, Leu116, Leu149, Lys49, Phe120, Pro45, Ser115, Thr44, Thr50, Thr147
8	Lapathoside B	−7.1	5/Arg78, Asn34, Asn36, Asn135, Asn137, Asn248, Asp35, Asp138, Gln80, Glu250, Ile37, Leu96, Leu132, Leu251, Lys116, Lys134, Met33, Phe39, Pro117, Ser38, Ser41, Ser115, Thr9, Thr82, Thr113, Tyr136, Val81, Val119	−6.1	4 */Arg127, Asp145, Br303, Br305, Gly46, Gly148, Ile119, Leu116, Leu149, Lys49, Phe120, Pro45, Thr44, Thr50, Thr147
9	Orientin	−6.2	4 */Asn34, Asn135, Asp35, Asp138, Leu132, Met33, Pro117, Ser115	−5.1	2 */Arg127, Br303, Gly46, Gly148, Leu149, Pro45
10	Quercetin_3-O-6″-(3-hydroxyl-3-methylglutaryl)-D-glucopyranoside	−7.6	5 */Arg78, Asn34, Asn135, Asn248, Gln80, Glu250, Leu96, Leu251, Lys116, Lys134, Met33, Pro117, Ser115, Thr113, Thr249, Val81	−6.9	5 */Ala42, Arg124, Asn76, Asp77, Gly41, Gly121, Ile119, Phe43, Phe120, Pro45, Pro100, Thr44, Val75, Val80,
11	Vanicoside B	−6.7	4/Asn34, Asn36, Asn135, Asn248, Gln80, Glu250, Leu96, Leu132, Lys116, Lys134, Met33, Phe39, Pro117, Ser41, Thr9, Tyr136, Val119	−6.2	3 */Arg127, Asp145, Br305, Gly46, Gly148, Ile119, Leu116, Leu149, Lys49, Phe120, Pro45, Thr44, Thr50, Thr147
12	Vitexin	−6.3	5/Asn34, Asn135, Asn248, Gln80, Glu32, Glu250, Leu132, Leu251, Lys116, Lys134, Met33, Pro117, Ser115, Val81, Val119	−5.2	4/Arg127, Gly46, Gly148, Ile119, Leu116, Leu149, Lys49, Pro45, Thr147
FAD		−7.4	6/Arg78, Asn34, Asn135, Asn137, Asn248, Asp35, Asp138, Gln80, Leu96, Leu132, Lys116, Lys134, Met33, Pro117	−6.1	5 */Ala42, Arg124, Arg127, Gly46, Ile119, Pro45, Thr44, Thr147
Ben				−4	3/Arg127, Gly46, Gly148, Leu149, Thr147

* Unfavorable bonds were subtracted from the no. of formed bonds. BA: binding affinity (Kcal/mol); AA: amino acid residue; FAD = flavin-adenine dinucleotide (reference ligand ID:2CDU); Ben = benzoic acid (reference ligand ID: 1HD2).

**Table 3:** In silico ADME–physicochemical predictions of the predominant compounds 1–12 identified in different *R. vesicarius* organs.

Predictive Parameters	1	2	3	4	5	6	7	8	9	10	11	12
ADME Prediction												
Physicochemical parameters												
TPSA (Å):	250.22 Å^2^	266.66 Å^2^	268.43 Å^2^	201.28 Å^2^	181.05 Å^2^	69.92 Å^2^	313.19 Å^2^	322.42 Å^2^	201.28 Å^2^	274.11 Å^2^	303.96 Å^2^	181.05 Å^2^
MR	86.06	173.62	192.27	108.63	106.61	118.59	246.63	253.12	108.63	142.09	240.14	106.61
Drug likeness Prediction												
Bioavailability Value	0.17				0.55		0.17					0.55
Synthetic accessibility	5.97	6.7	6.86	5.04	4.99	4.34	7.95	8.12	5.17	6.1	7.77	5.12
Absorption Prediction												
Log *S* (ESOL)	1.55	−3.2	−5.01	−2.7	−2.84	−6.42	−6.97	−7.07	−2.7	−2.84	−6.88	−2.84
Consensus Log *P_o_*_/*w*_	−4.82	−0.3	1.5	−0.24	−0.24	5.11	2.61	2.61	−0.41	−0.57	−0.57	−0.07
Solubility class	Highly soluble	Soluble	Moderately soluble	Soluble	Soluble	Poorly soluble	Poorly soluble	Poorly soluble	Soluble	Soluble	Poorly soluble	Soluble
Pharmacokinetics												
Log *Kp* (skin permeation, cm/s)	−13.01	−11.08	−9.69	−9.14	−8.79	−4.02	−9.75	−9.95	−9.14	−10.45	−9.55	−8.79
GI absorption	Low					High	Low					
BBB permeant	No											
Metabolism Estimation												
P-gp substrate	Yes	Yes	Yes	No	No	No	Yes	Yes	No	Yes	Yes	No
CYP1A2, CYP2C19, CYP2C9, CYP2D6, and CYP3A4 inhibitors	No					All No, except Yes (CYP3A4, CYP2C9)	No					

MR: molar refractivity, “Å^2^” polar surface area, “TPSA” Topological polar surface area, and Gentiobiosylglycerol (1), Helonioside B (2), Hydropiperoside (3), Isoorientin (4), Isovitexin (5), Kazinol A (6), and Lapathoside A (7), Lapathoside B (8), Orientin (9), Quercetin-3-*O*-6″-(3-hydroxyl-3-methylglutaryl)-D-glucopyranoside (10), Vanicoside B (11), Vitexin (12). SwissADME web tool [55].

## Data Availability

All data are within the manuscript and Supplementary Materials.

## Supplementary Materials

The following supporting information can be downloaded at: https://www.mdpi.com/article/10.3390/plants13131815/s1, Table S1. IC_50_ for polar fractions of leaves, flowers, roots, stems extracts and ascorbic acid as std against DPPH, ABTS, H_2_O_2_, FRAP, and TAC scavenging activities, Results are shown as means ± S.D. (measured in triplicate; n = 3). Means in the same column that do not share a letter are significantly different (*p* < 0.05) using ANOVA followed by Tukey as a post-hoc test. Table S2. DPPH scavenging % activity (IC_50_ value) of four plant parts extract at different concentration. Table S3. ABTS scavenging % activity (IC_50_ value) of four plant parts extract at different concentration. Table S4. H_2_O_2_ scavenging % activity (IC_50_ value) of four plant parts extract at different concentration. Table S5. FRAP scavenging % activity (IC_50_ value) of four plant parts extract at different concentration. Table S6. TAC scavenging % activity (IC_50_ value) of four plant parts extract at different concentration. Figure S1: Evaluation of Antioxidant Activity using DPPH scavenging. Figure S2: Evaluation of Antioxidant Activity using ABTS radical Scavenging. Figure S3: Evaluation of Antioxidant Activity using H_2_ O_2_ Scavenging Assay. Figure S4: Evaluation of Antioxidant Activity using FRAP scavenging %, Figure S5. Evaluation of Antioxidant Activity using TAC scavenging %. Figure S6. Representative molecular interactions (2D and 3D) of the most dominant chemical compounds (**1**–**12**), **1**: Gentiobiosylglycerol, **2**: Helonioside B, **3**: Hydropiperoside, **4**: Isoorientin, **5**: Isovitexin, **6**: Kazinol A, **7**: Lapathoside A, **8**: Lapathoside B, **9**: Orientin, **10**: Quercetin-3-*O*-6″-(3-hydroxyl-3-methylglutaryl)-D-glucopyranoside, **11**: Vanicoside B, and **12**: Vitexin, identified in different *R. vesicarius* organs with **A:** NADPH oxidase (NO) receptor (PDB ID:2CDU, 1.80Å, and **B:** human peroxiredoxin 5 enzyme (PDB code: 1HD2, 1.50 Å), 13: FAD (Flavin-adenine dinucleotide) as a co-crystallized ligand.
