# Supplementary material for: Unravelling the Phytochemical Composition and Antioxidant Potential of Different Parts of Rumex vesicarius L.: A RP-HPLC-MS-MS/MS, Chemometrics, and Molecular Docking-Based Comparative Study
Source: Plants (Basel). 2024 Jul 1;13(13):1815. doi: 10.3390/plants13131815 (PMC11244572; doi:10.3390/plants13131815)
Supplement: Supplementary file 1 [file plants-13-01815-s001.zip › plants-3069870-supplementary.pdf]

Table S1. IC_{50} for of leaves, flowrs, roots, stems extracts and ascorbic acid as standard against DPPH, ABTS, H_2O_2, FRAP, and TAC scavenging activities, Results are shown as means ± S.D. (measured in triplicate; n = 3). Means in the same column that do not share a letter are significantly different ($p < 0.05$) using ANOVA followed by Tukey as a post-hoc test.

	**DPPH%**	**ABTS scavenging %**	**H_2O_2 scavenging %**	**FRAP scavenging %**	**TAC scavenging %**
**Leaves**	209.24 ± 11.15 [b,c,d,e]	157.82 ± 15.61 [b,c,d,e]	231.27 ± 7.38 [b,c,d,e]	352.59 ± 29.62 [b,c,d,e]	145.67 ± 8.64 [b,c,d,e]
**Flowers**	17.93 ± 1.81 [a]	14.06 ± 0.54 [a]	28.08 ± 1.45 [a,c,d,e]	39.47 ± 8.88 [a]	12.02 ± 0.24 [a]
**Roots**	28.94 ± 0.77 [a,d]	25.68 ± 1.22 [a]	41.55 ± 4 [a,b,d,e]	58.03 ± 1.61 [a,d,e]	21.6 ± 4.39 [a,e]
**Stems**	14.11 ± 0.52 [a,c]	12.28 ± 0.47 [a]	14.99 ± 0.11 [a,b,c]	21.37 ± 4.01 [a,c]	10.16 ± 0.77 [a]
**Ascorbic acid**	10.21 ± 0.77 [a,c]	10.66 ± 0.89 [a]	14.77 ± 0.69 [a,b,c]	20.89 ± 1.25 [a,c]	7.12 ± 0.95 [a,c]

Table S2. DPPH scavenging % activity (IC50 value) of four plant parts extract at different concentration

DPPH scavenging %

**Sample conc. (µg/ml)**	**Leaves**		**Flowers**		**Roots**		**Stems**		**Std**	
	Mean	S.D. (±)	Mean	S.D. (±)	Mean	S.D. (±)	Mean	S.D. (±)	Mean	S.D. (±)
**1000**	72.08	1.61	91.07	0.53	85.62	1	95.77	0.36	98.65	0.1
**500**	64.17	1.95	86.84	1.41	80.15	1.28	91.32	0.6	96.34	0.65
**250**	56.6	2.16	80.36	0.43	74.25	1.87	85.45	0.74	94.25	0.92
**125**	36.54	1.14	75.8	0.58	69.6	0.96	79.74	1.25	90.89	0.17
**62.5**	25.16	1.26	69.29	1.12	61.7	0.88	71.84	1.94	86.42	0.71
**31.25**	18.08	1.97	60.25	1.83	52.02	0.63	62.35	2.19	79.35	1.22
**15.6**	10.65	1.49	48.23	1.32	37.95	1.08	53	1.15	65.05	0.21
**7.8**	6.81	1.43	37.56	1.69	28.33	1.33	37.41	1.43	43.01	2.87
**3.9**	5.32	0.91	25.88	1.27	21.76	1.01	29.87	2.2	31.53	1.04
**2**	3.97	0.81	19.41	1.12	15.75	1.14	23.34	1.56	22.18	1.37
**1**	2.82	0.36	13.12	1.26	10.64	0.67	16.64	0.6	14.48	0.62
**0.5**	1.46	0.21	8.34	0.58	5.99	0.19	10.94	0.53	9.34	0.46
**IC_{50}**	**209.24**	**11.15**	**17.93**	**1.81**	**28.94**	**0.77**	**14.11**	**0.52**	**10.21**	**0.77**

Table S3. ABTS scavenging % activity (IC50 value) of four plant parts extract at different concentration

	ABTS scavenging %									
**Sample conc. (μg/ml)**	**Leaves**		**Flowers**		**Roots**		**Stems**		**Std**	
	Mean	S.D. (±)	Mean	S.D. (±)	Mean	S.D. (±)	Mean	S.D. (±)	Mean	S.D. (±)
**1000**	76.38	0.62	91.84	0.26	87.55	1.09	96.04	0.08	95.46	2.24
**500**	69.11	1.83	87.5	0.84	81.25	0.97	92.43	0.27	95.3	1.31
**250**	61.14	2.1	81.85	0.43	75.81	1.14	88.07	0.42	93.05	0.49
**125**	46.05	1.84	76.26	1.12	70.1	1.21	80.95	1.39	91.29	0.76
**62.5**	36.11	0.82	70.02	1.09	62.97	1.15	73.81	1.39	85.22	0.99
**31.25**	28.77	0.8	62.17	1.48	54.79	1.3	66.57	1.57	72.28	2.52
**15.6**	20.52	0.83	52.36	1.2	41.35	0.71	56.23	0.94	62.36	1.69
**7.8**	13.36	1.55	40.67	2.2	33.99	1.18	41.61	0.9	42.39	3.66
**3.9**	8.91	0.84	32.05	1.59	27.14	0.48	33.22	1.74	33.1	1.22
**2**	6.04	0.84	22.8	2.02	20.61	0.87	24.57	1.51	24.98	1.39
**1**	4.02	0.32	15.18	1.15	12.98	2.29	17.94	1.39	18.13	2.26
**0.5**	2.57	0.25	9.11	0.55	7.61	1.2	13.35	1.42	10.87	1.47
**IC_{50}**	**157.82**	**15.61**	**14.06**	**0.54**	**25.68**	**1.22**	**12.28**	**0.47**	**10.66**	**0.89**

Table S4. H_2O_2 scavenging % activity (IC50 value) of four plant parts extract at different concentration

	H_2O_2 scavenging %									
**Sample conc. (μg/ml)**	**Leaves**		**Flowers**		**Roots**		**Stems**		**Std**	
	Mean	S.D. (±)	Mean	S.D. (±)	Mean	S.D. (±)	Mean	S.D. (±)	Mean	S.D. (±)
**1000**	68.54	1.49	88.13	0.61	84.75	0.41	94.16	0.48	95.73	0.68
**500**	60.11	1.79	82.41	1.12	78.24	1.35	88.92	0.44	92.55	1.19
**250**	52.43	0.9	75.16	0.94	71.18	0.62	83.62	1.73	89.91	1.09

**125**	36.04	1.61	68.83	1.53	64.4	1.13	76.5	0.64	83.72	1.14
**62.5**	26.44	3.69	60.89	1.89	57.78	2.27	70.47	0.61	73.42	2.77
**31.25**	17.34	1.32	52.77	1.24	46.02	1.96	62.57	0.57	63.3	1.68
**15.6**	10.66	0.81	38.86	1.93	38.25	2.23	51.17	0.27	51.79	1.44
**7.8**	7.39	0.81	30.97	1.75	26.22	1.64	36.4	0.58	34.7	2.02
**3.9**	5.14	0.73	25.11	1.63	19.03	1.84	25.59	0.59	25.36	0.65
**2**	3.36	0.46	18.4	2.13	11.88	0.85	19.1	0.63	18.43	0.87
**1**	2.04	0.41	12.59	1.22	7.9	0.68	12.28	1.82	11.73	1.24
**0.5**	1.04	0.19	7.09	0.77	4.19	0.56	7.98	1.47	6.2	0.48
**IC $_{50}$**	**231.27**	**7.38**	**28.08**	**1.45**	**41.55**	**4**	**14.99**	**0.11**	**14.77**	**0.69**

Table S5. FRAP scavenging % activity (IC50 value) of four plant parts extract at different concentration

FRAP scavenging %

**Sample conc. (μg/ml)**	**Leaves**		**Flowers**		**Roots**		**Stems**		**Std**	
	Mean	S.D. (±)	Mean	S.D. (±)	Mean	S.D. (±)	Mean	S.D. (±)	Mean	S.D. (±)
**1000**	64.08	1.06	87.22	1.11	78.74	1.69	93.68	92.79	94.19	0.38
**500**	55.6	1.13	80.15	1.67	72.62	1.37	88.17	87.05	92.87	0.21
**250**	46.08	1.24	73.34	1.59	64.38	1.04	81.95	80.42	90.57	1.17
**125**	33.73	1.47	64.69	2.57	57.63	1.72	74.69	71.96	82.95	1.09
**62.5**	23.45	1.38	56.76	2.43	51.36	0.73	65.94	63.18	71.2	1.88
**31.25**	14.61	0.75	47.37	3.15	42.2	1.35	58.17	56.03	59.92	1.61
**15.6**	9.19	0.29	33.88	2.61	35.06	1.32	49.26	45.14	44.93	1.15
**7.8**	6.03	0.65	26.81	1.86	27.4	1.73	36.45	31.78	34.15	1.43
**3.9**	4.02	0.14	19.92	1.49	20.85	0.55	28.16	26.41	25.9	1.21
**2**	2.81	0.4	13.73	2.53	14.44	0.72	20.43	19.86	18.89	1.46
**1**	1.85	0.11	9.31	0.98	9.45	0.9	15.39	14.97	12.46	1.56
**0.5**	0.99	0.04	5.98	0.29	5.37	0.6	9.76	8.64	7.16	1.31
**IC $_{50}$**	**352.59**	**29.62**	**39.47**	**8.88**	**58.03**	**1.61**	**21.37**	**4.01**	**20.89**	**1.25**

Table S6. TAC scavenging % activity (IC50 value) of four plant parts extract at different concentration

	TAC scavenging %									
**Sample conc. (μg/ml)**	**Leaves**		**Flowers**		**Roots**		**Stems**		**Std**	
	Mean	S.D. (±)	Mean	S.D. (±)	Mean	S.D. (±)	Mean	S.D. (±)	Mean	S.D . (±)
**1000**	76.18	1.09	92.89	0.64	88.45	0.51	96.81	0.34	99.34	1.09
**500**	69.23	1.17	89.08	0.48	84.36	0.6	93.16	0.26	96.45	0.64
**250**	61.78	1.23	84.59	0.46	78.66	1.09	89.34	1.1	94.65	0.34
**125**	47.63	1.14	77.15	0.93	71.44	0.85	85.31	0.63	89.77	1.32
**62.5**	38.86	1.36	71.32	0.78	65.37	1.54	80.79	1.52	85.45	2.34
**31.25**	30.08	1.74	66.23	1.18	56.89	3.2	71.22	0.79	75.55	1.2
**15.6**	22.64	1.03	55.46	1.2	45.35	4.13	61.64	2.85	60.38	0.75
**7.8**	15.04	1.72	43.6	1.16	36.73	1.88	45.08	1.29	55.23	0.55
**3.9**	10.03	0.83	36.25	1.16	29.95	1.28	34.89	2.55	40.24	0.84
**2**	6.15	0.29	28.97	2.22	23.83	1.83	29.92	0.72	35.34	0.98
**1**	3.75	0.26	22.16	2.35	18.18	1.45	24.22	1.13	30.67	2.65
**0.5**	2.13	0.28	15.44	1.2	11.41	0.93	19.05	1.41	22.34	1.23
**IC_{50}**	**145.67**	**8.64**	**12.02**	**0.24**	**21.6**	**4.39**	**10.16**	**0.77**	**7.12**	**0.95**

Figure S1: Evaluation of Antioxidant Activity using DPPH scavenging

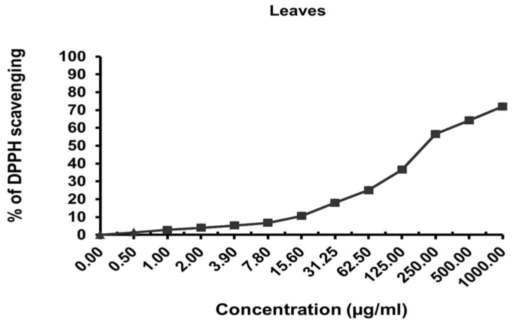

A

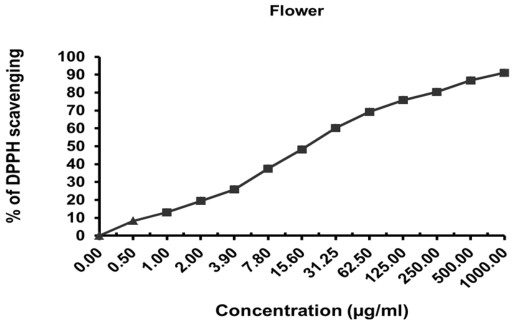

B

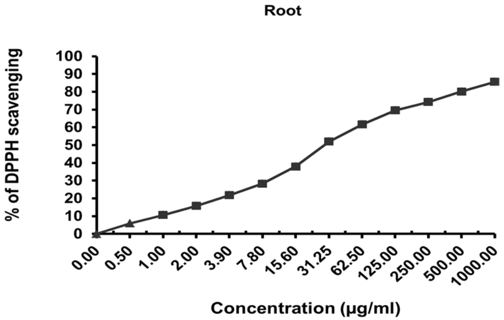

C

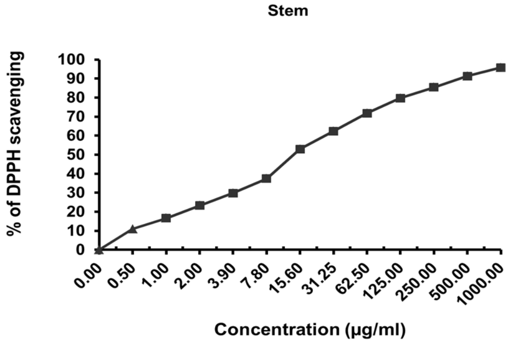

D

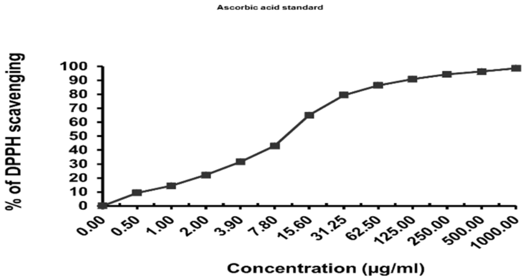

E (Ascorbic acid)

Figure S2: Evaluation of Antioxidant Activity using ABTS radical Scavenging

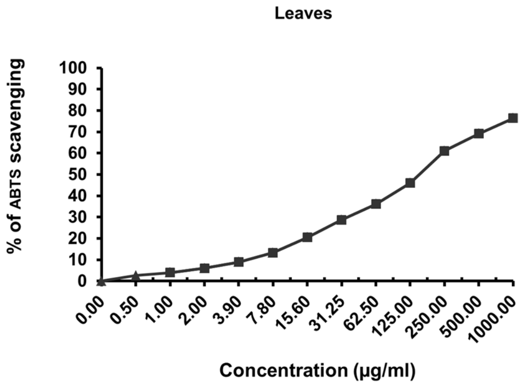

A

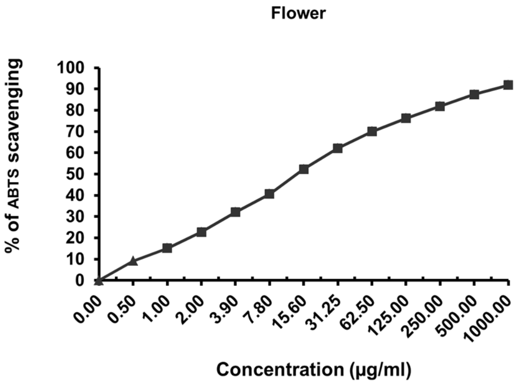

B

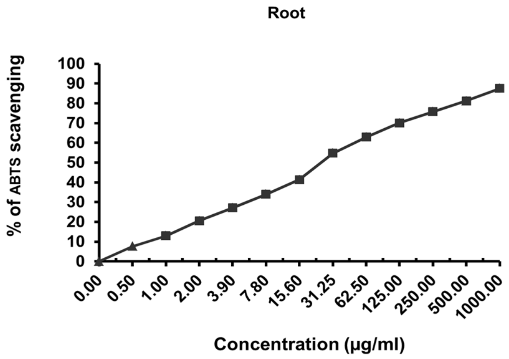

C

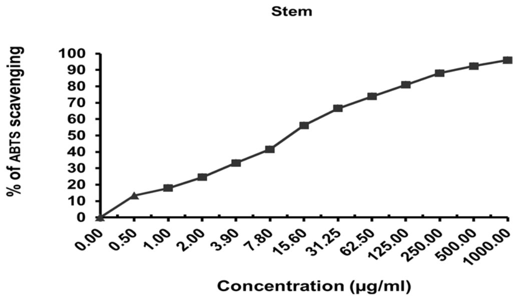

D

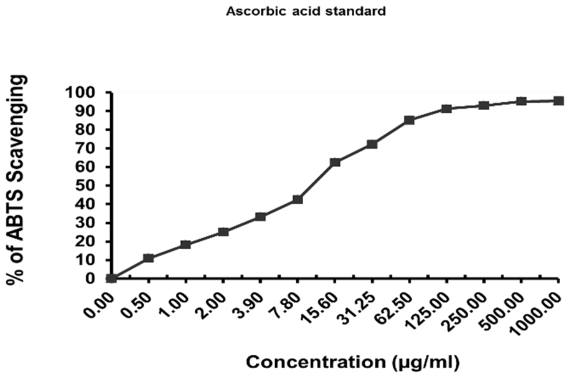

E (Ascorbic acid)

Figure S3: Evaluation of Antioxidant Activity using H_2O_2 Scavenging Assay

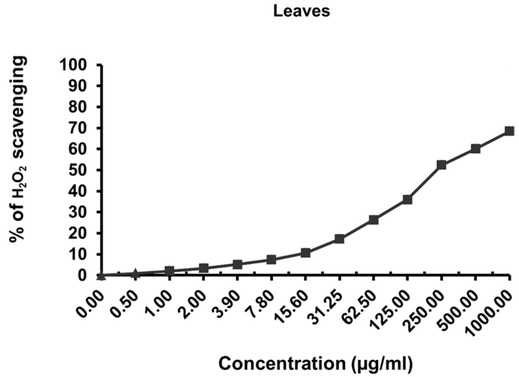

A

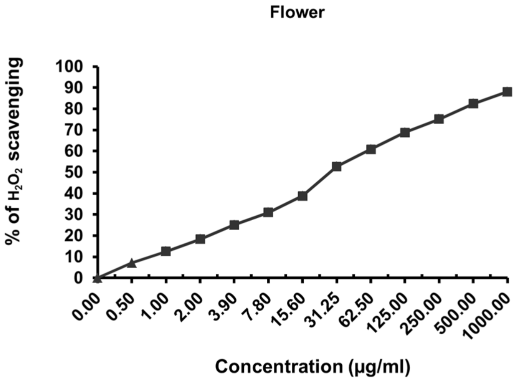

B

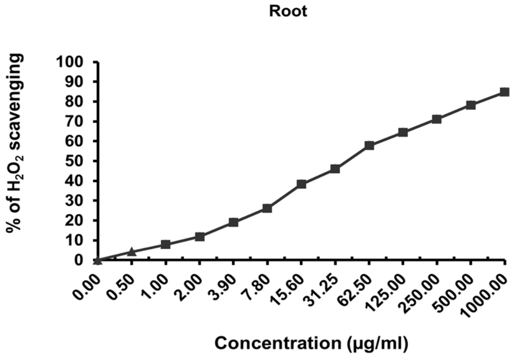

C

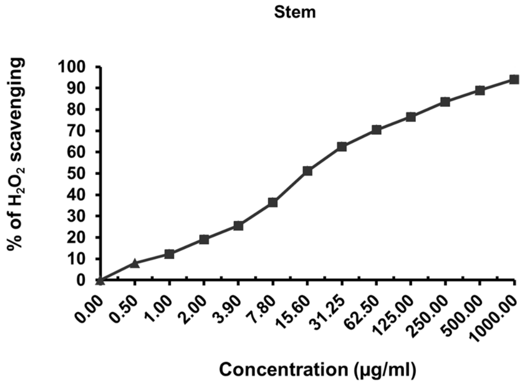

D

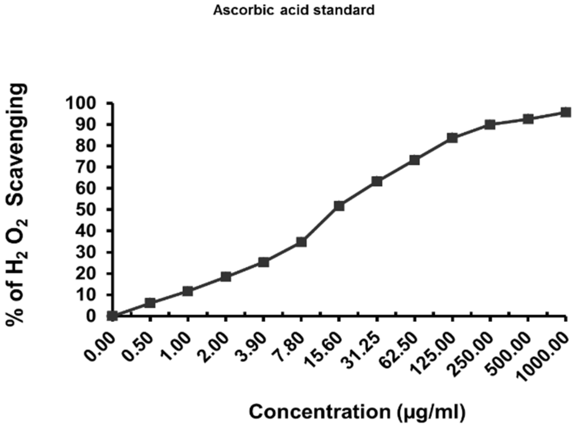

E (Ascorbic acid)

Figure S4: Evaluation of Antioxidant Activity using FRAP scavenging %

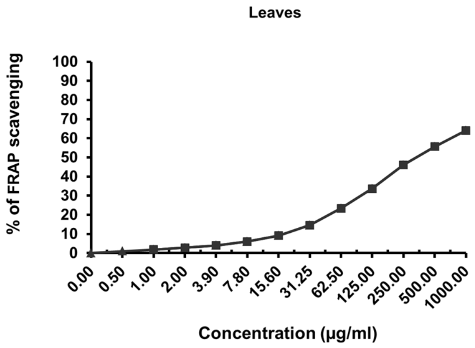

A

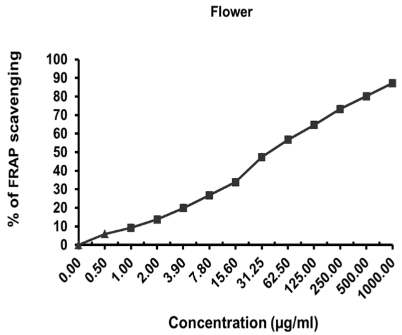

B

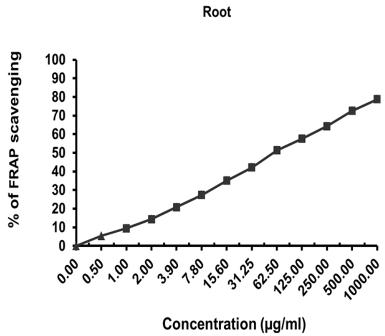

C

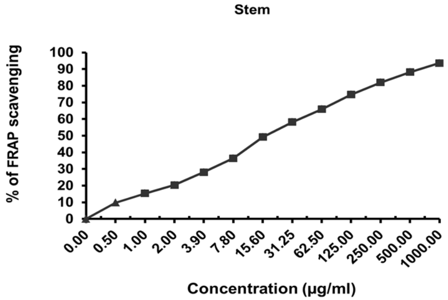

D

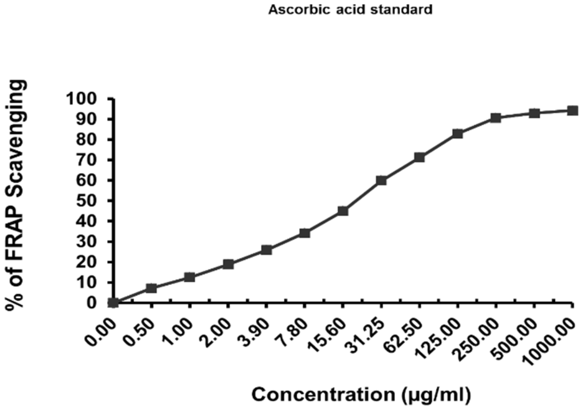

E (Ascorbic acid)

Figure S5. Evaluation of Antioxidant Activity using TAC scavenging %

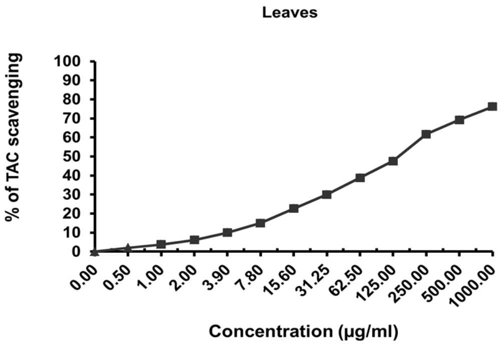

A

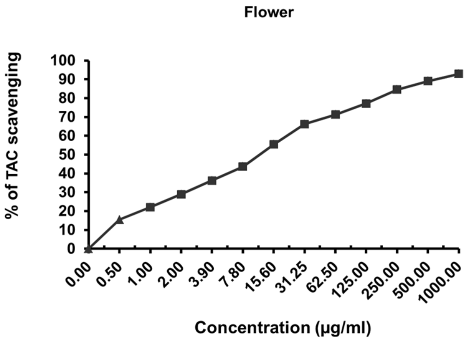

B

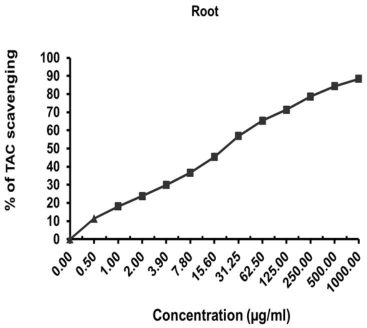

C

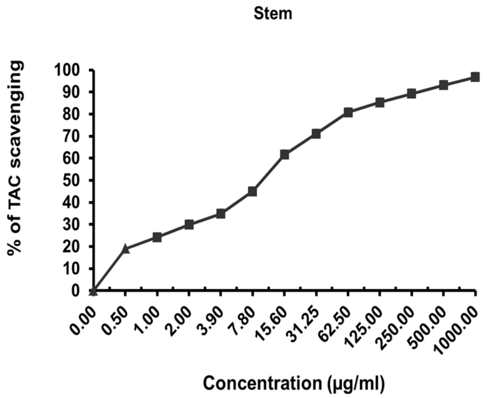

D

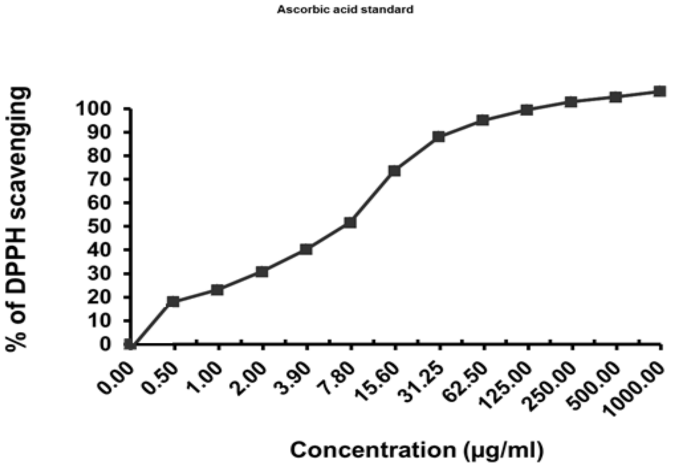

E (Ascorbic acid)

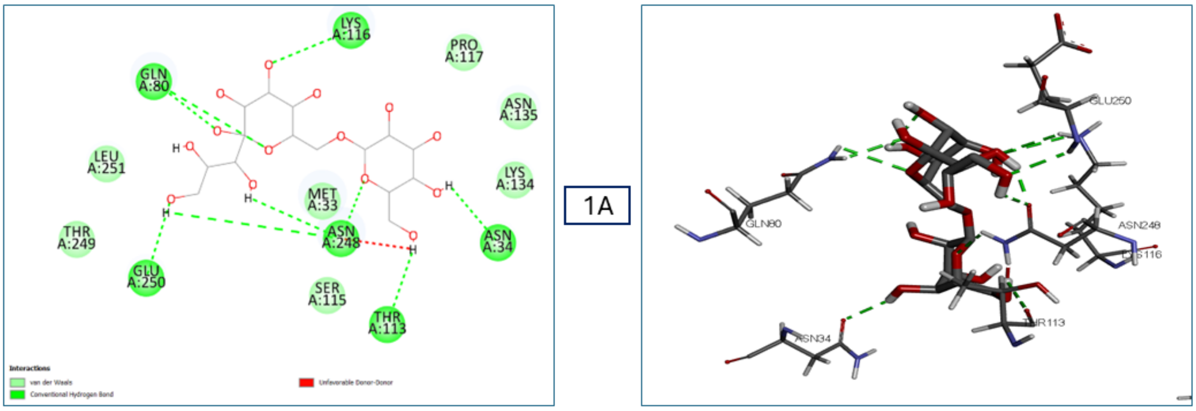
LYS A:116
PRO A:117
GLN A:80
ASN A:135
LEU A:251
LYS A:134
MET A:33
THR A:249
ASN A:248
ASN A:34
GLU A:250
SER A:115
THR A:113
Interactions
van der Waals
Conventional Hydrogen Bond
Unfavorable Donor-Donor
GLU250
GLN80
ASN248
LYS116
ASN34
THR113

1A

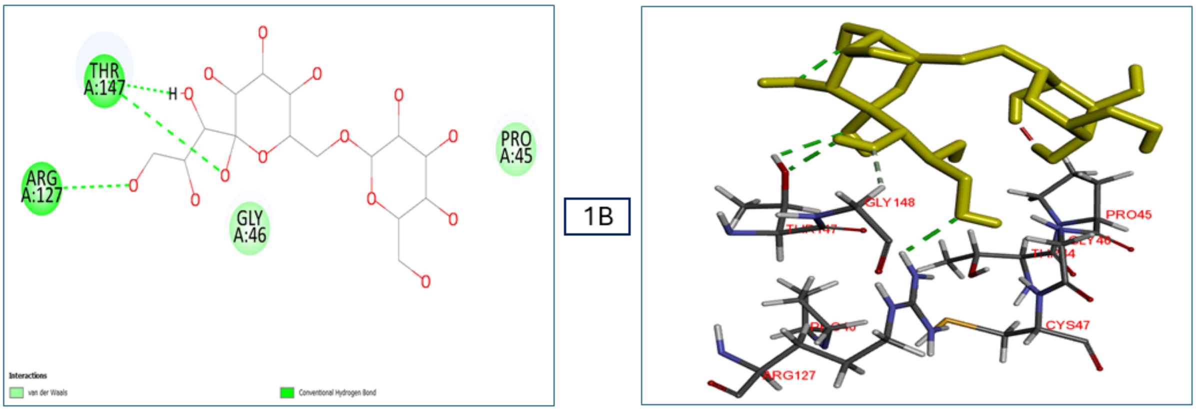
THR A:147
PRO A:45
ARG A:127
GLY A:46
Interactions
van der Waals
Conventional Hydrogen Bond
GLY148
PRO45
GLY46
CYS47
ARG127

1B

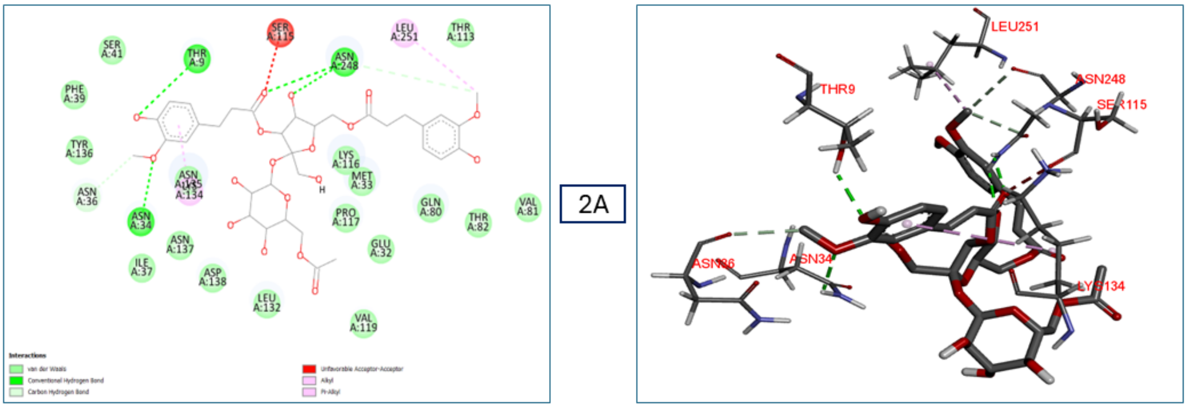
SER A:115
LEU A:251
THR A:113
SER A:41
THR A:9
ASN A:248
PHE A:39
TYR A:136
LYS A:116
ASN A:36
MET A:33
ASN A:34
PRO A:117
GLN A:80
THR A:82
VAL A:81
ASN A:137
GLU A:32
ILE A:37
ASP A:138
LEU A:132
VAL A:119
Interactions
van der Waals
Conventional Hydrogen Bond
Carbon Hydrogen Bond
Unfavorable Acceptor-Acceptor
Alkyl
Pi-Alkyl
LEU251
THR9
ASN248
ASN36
ASN34
LYS134

2A

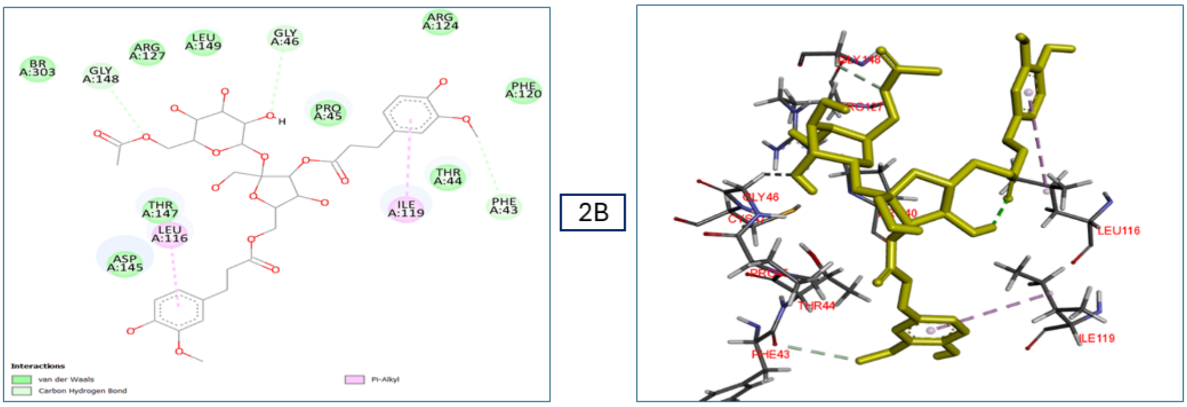
ARG A:124
BR A:303
ARG A:127
LEU A:149
GLY A:46
GLY A:148
PHE A:120
PRO A:45
THR A:44
THR A:147
ILE A:119
PHE A:43
LEU A:116
ASP A:145
Interactions
van der Waals
Carbon Hydrogen Bond
Pi-Alkyl
GLY46
LEU116
THR44
PHE43
ILE119

2B

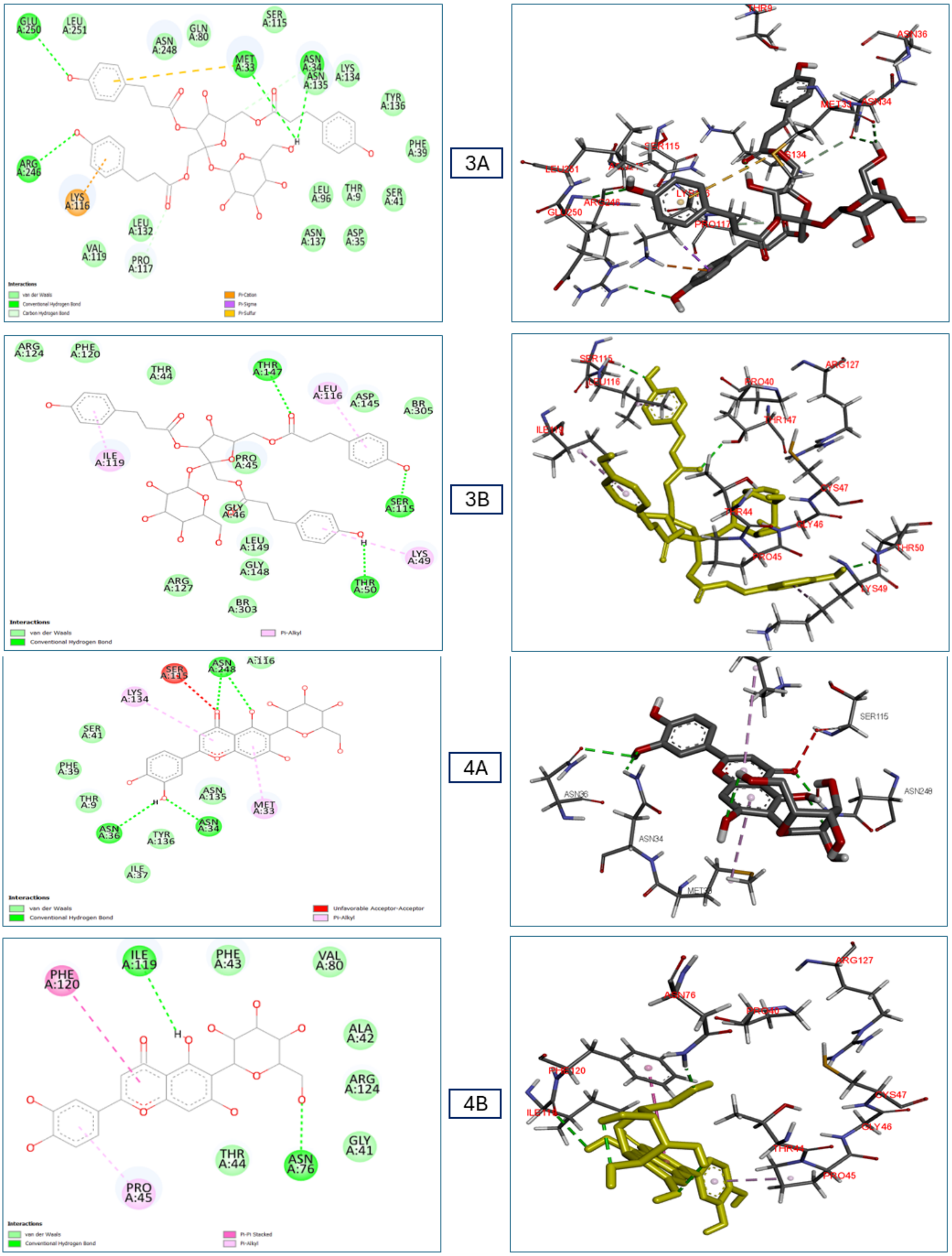
3A
3B
4A
4B

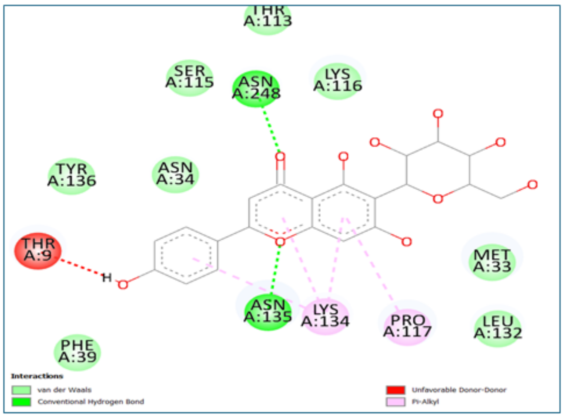
THR A:113
SER A:115
ASN A:248
LYS A:116
TYR A:136
ASN A:34
THR A:9
MET A:33
ASN A:135
LYS A:134
PRO A:117
LEU A:132
PHE A:39
Interactions
van der Waals
Conventional Hydrogen Bond
Unfavorable Donor-Donor
Pi-Alkyl

5A

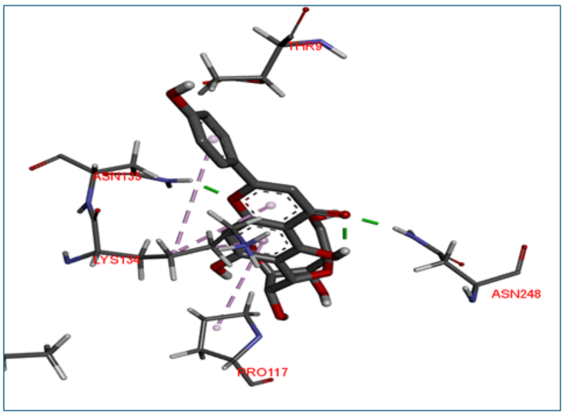
ASN248

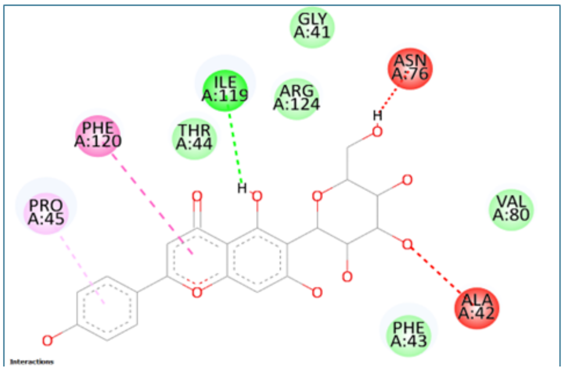
GLY A:41
ASN A:76
ILE A:119
ARG A:124
PHE A:120
THR A:44
PRO A:45
VAL A:80
ALA A:42
PHE A:43
Interactions

5B

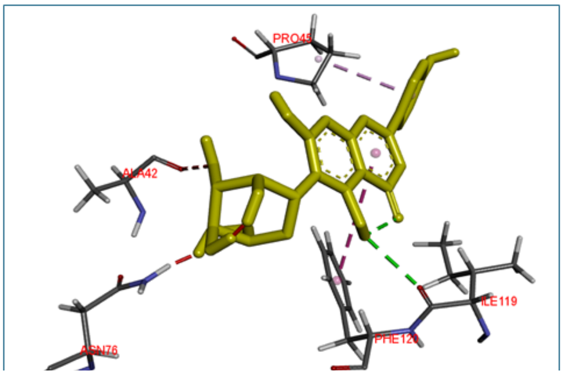
ILE119

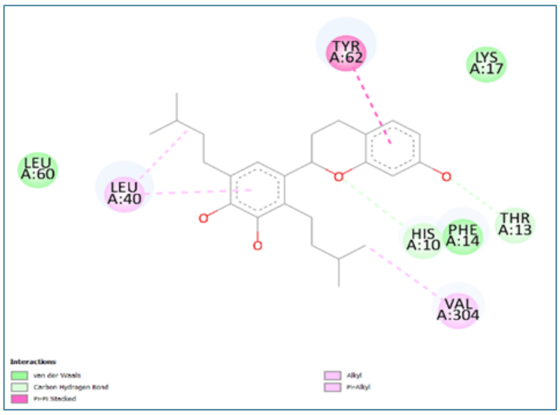
TYR A:62
LYS A:17
LEU A:60
LEU A:40
HIS A:10
PHE A:14
THR A:13
VAL A:304
Interactions
van der Waals
Carbon Hydrogen Bond
Pi-Pi Stacked
Alkyl
Pi-Alkyl

6A

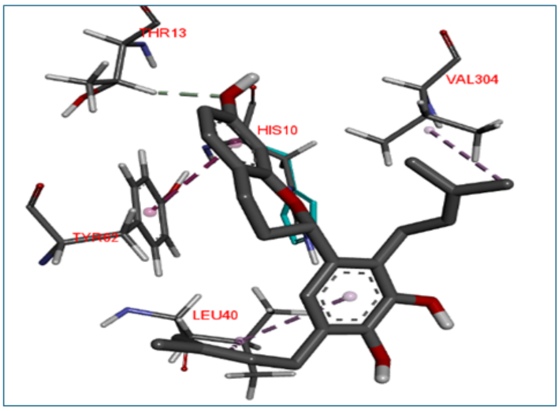
THR13
VAL304
HIS10
TYR62
LEU40

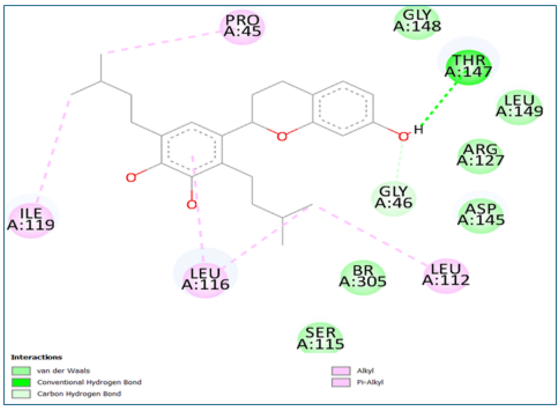
PRO A:45
GLY A:148
THR A:147
LEU A:149
ARG A:127
GLY A:46
ASP A:145
ILE A:119
LEU A:116
BR A:305
LEU A:112
SER A:115
Interactions
van der Waals
Conventional Hydrogen Bond
Carbon Hydrogen Bond
Alkyl
Pi-Alkyl

6B

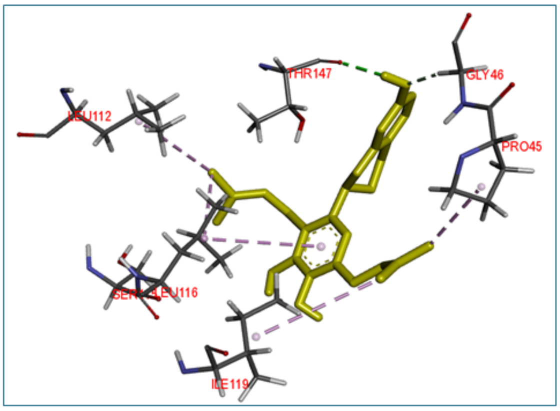
THR147
GLY46
LEU112
PRO45
ILE119

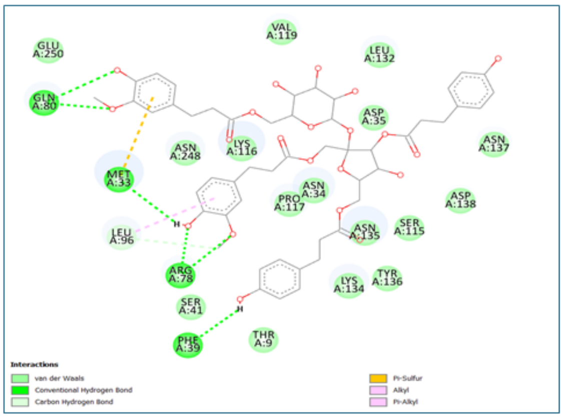
GLU A:250
VAL A:119
LEU A:132
GLN A:80
ASP A:35
ASN A:137
LYS A:116
ASN A:248
MET A:33
ASN A:34
PRO A:117
ASP A:138
LEU A:96
ASN A:135
SER A:115
ARG A:78
LYS A:134
TYR A:136
SER A:41
PHE A:39
THR A:9
Interactions
van der Waals
Conventional Hydrogen Bond
Carbon Hydrogen Bond
Pi-Sulfur
Alkyl
Pi-Alkyl

7A

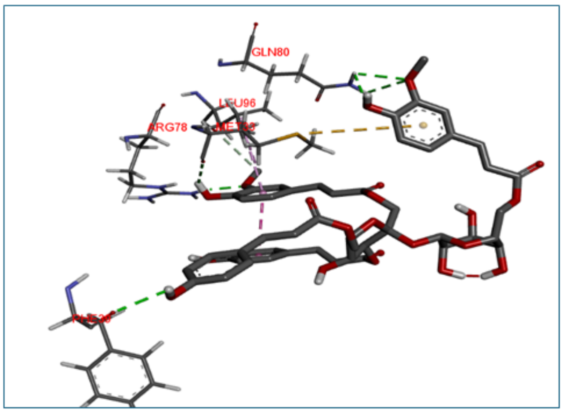
GLN80
LEU96
ARG78
MET33

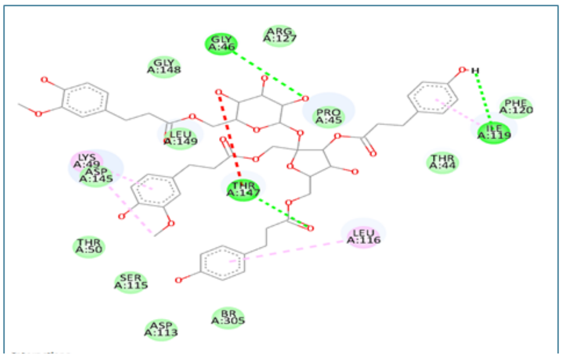
GLY A:46
ARG A:127
GLY A:148
PRO A:45
PHE A:120
LEU A:149
ILE A:119
LYS A:49
ASP A:145
THR A:44
THR A:147
LEU A:116
THR A:50
SER A:115
BR A:305
ASP A:113

7B

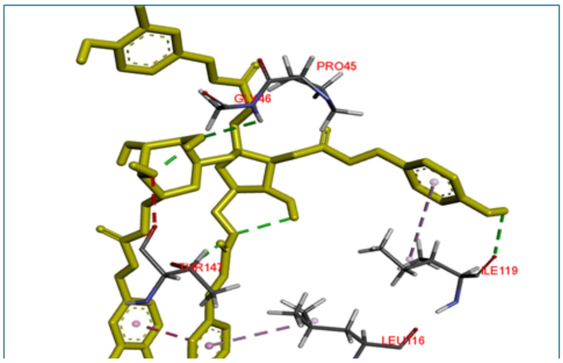
PRO45
GLY46
THR147
ILE119
LEU116

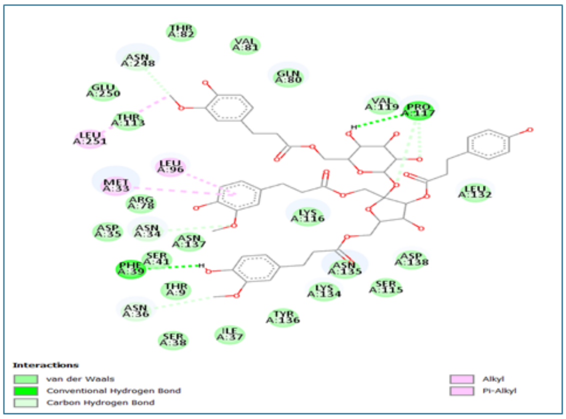
THR A:82
VAL A:81
ASN A:248
GLN A:80
GLU A:250
THR A:113
LEU A:251
VAL A:119
PRO A:117
LEU A:96
MET A:33
ARG A:78
LEU A:132
LYS A:116
ASP A:35
ASN A:34
ASN A:137
SER A:41
PHE A:39
ASN A:135
ASP A:138
THR A:9
LYS A:134
SER A:115
ASN A:36
TYR A:136
SER A:38
ILE A:37
Interactions
van der Waals
Conventional Hydrogen Bond
Carbon Hydrogen Bond
Alkyl
Pi-Alkyl

8A

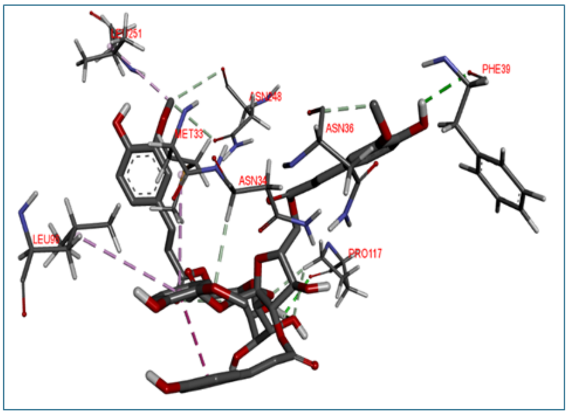
LEU251
ASN248
MET33
ASN36
PHE39
ASN34
PRO117

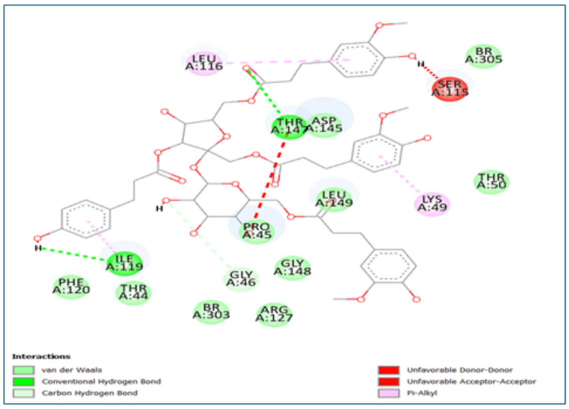
LEU A:116
BR A:305
SER A:115
THR A:147
ASP A:145
THR A:50
LEU A:149
LYS A:49
PRO A:45
ILE A:119
GLY A:46
GLY A:148
PHE A:120
THR A:44
BR A:303
ARG A:127
Interactions
van der Waals
Conventional Hydrogen Bond
Carbon Hydrogen Bond
Unfavorable Donor-Donor
Unfavorable Acceptor-Acceptor
Pi-Alkyl

8B

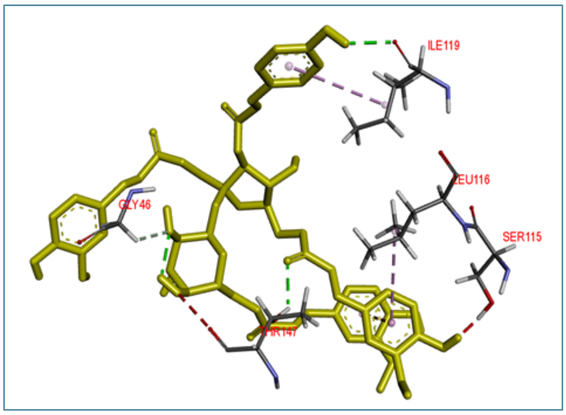
ILE119
LEU116
GLY46
SER115
THR147

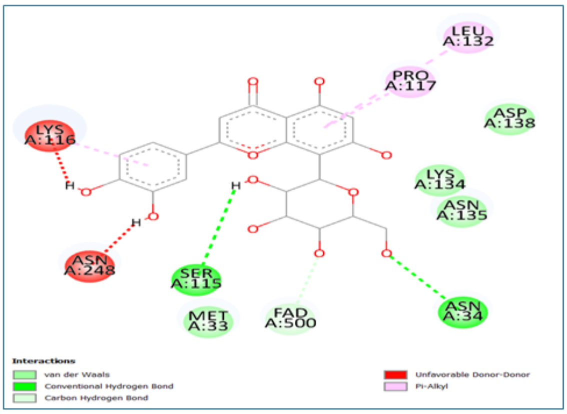
LEU A:132
PRO A:117
ASP A:138
LYS A:116
LYS A:134
ASN A:135
ASN A:248
SER A:115
MET A:33
FAD A:500
ASN A:34
Interactions
van der Waals
Conventional Hydrogen Bond
Carbon Hydrogen Bond
Unfavorable Donor-Donor
Pi-Alkyl

9A

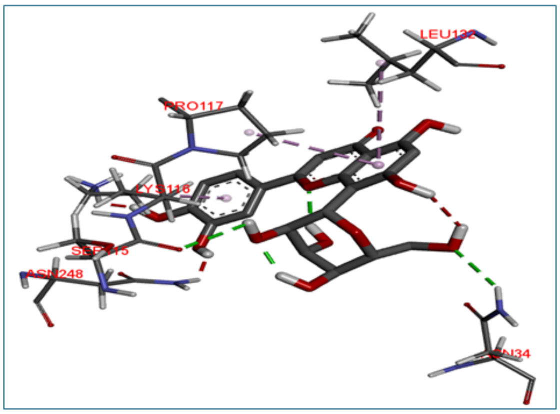
PRO117

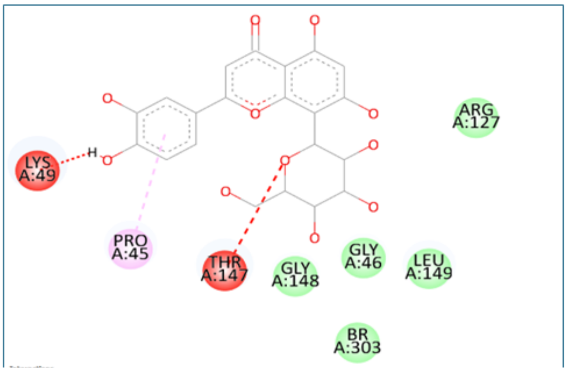
ARG A:127
LYS A:49
PRO A:45
THR A:147
GLY A:148
GLY A:46
LEU A:149
BR A:303

9B

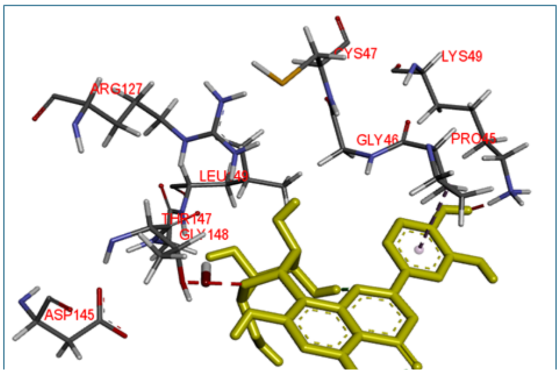
LYS49
ARG127
GLY46
THR147
GLY148
ASP145

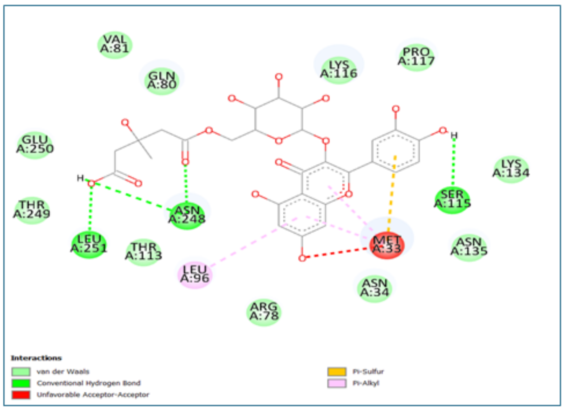
VAL A:81
GLN A:80
LYS A:116
PRO A:117
GLU A:250
LYS A:134
THR A:249
SER A:115
ASN A:248
LEU A:251
THR A:113
MET A:33
ASN A:135
LEU A:96
ASN A:34
ARG A:78
Interactions
van der Waals
Conventional Hydrogen Bond
Unfavorable Acceptor-Acceptor
Pi-Sulfur
Pi-Alkyl

10A

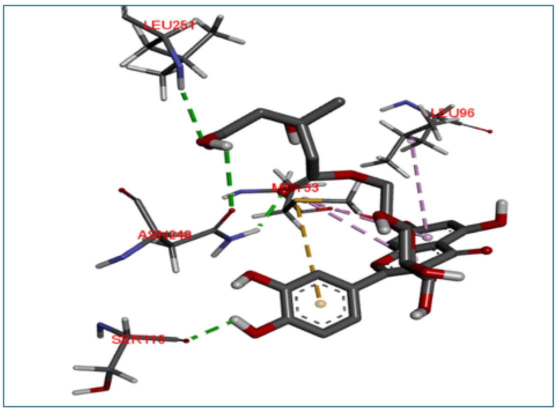
LEU251

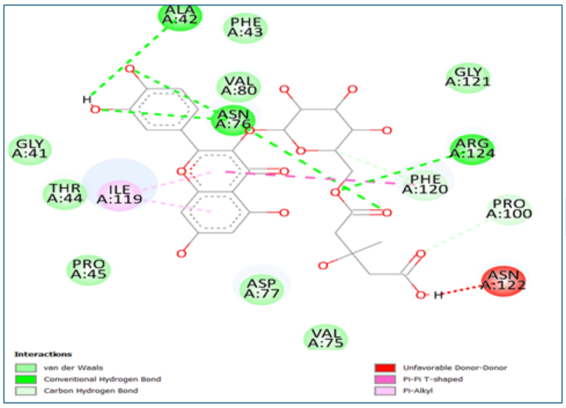
ALA A:42
PHE A:43
VAL A:80
GLY A:121
ASN A:76
GLY A:41
ARG A:124
PHE A:120
THR A:44
ILE A:119
PRO A:100
PRO A:45
ASP A:77
ASN A:122
VAL A:75
Interactions
van der Waals
Conventional Hydrogen Bond
Carbon Hydrogen Bond
Unfavorable Donor-Donor
Pi-Pi T-shaped
Pi-Alkyl

10B

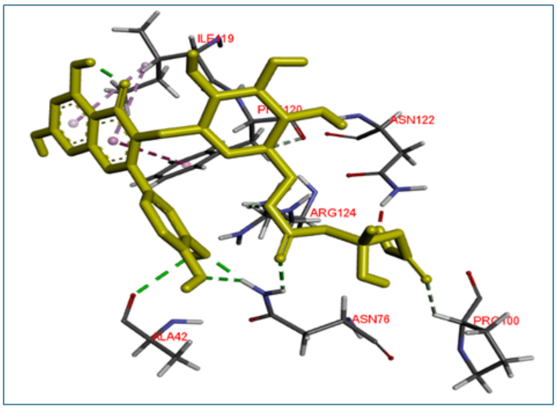
ASN122
ARG124
ASN76
ALA42

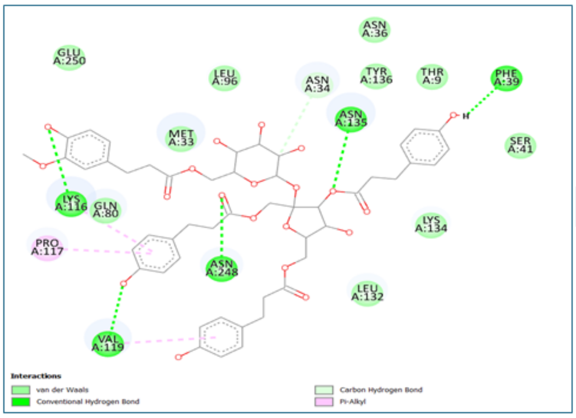

11A

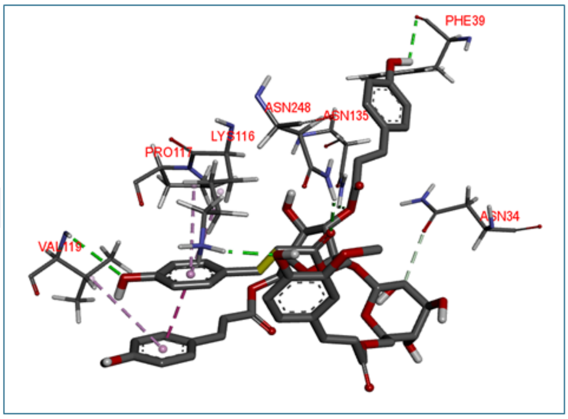

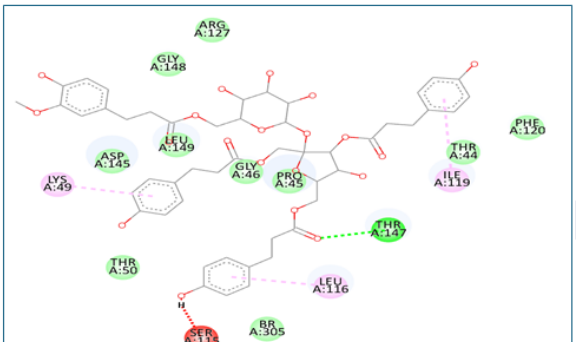

11B

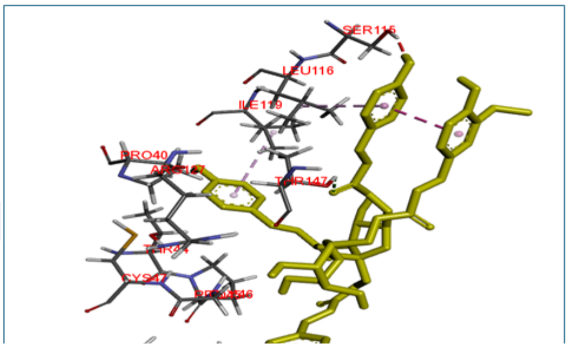

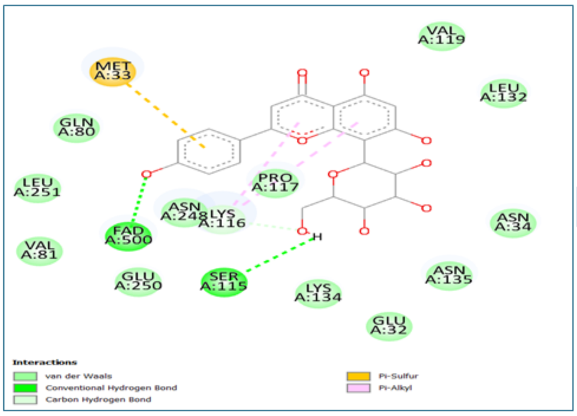

12A

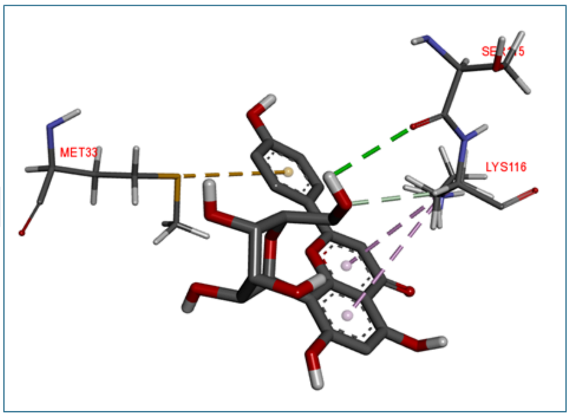

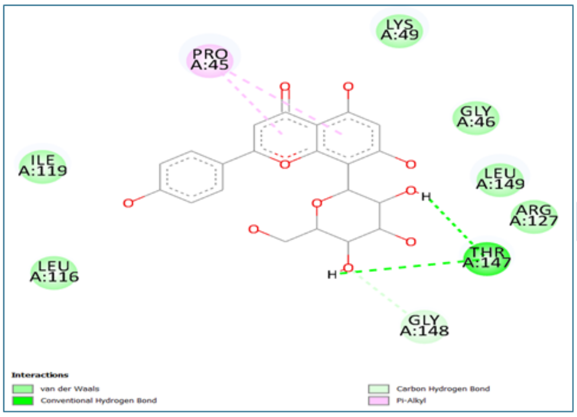

12B

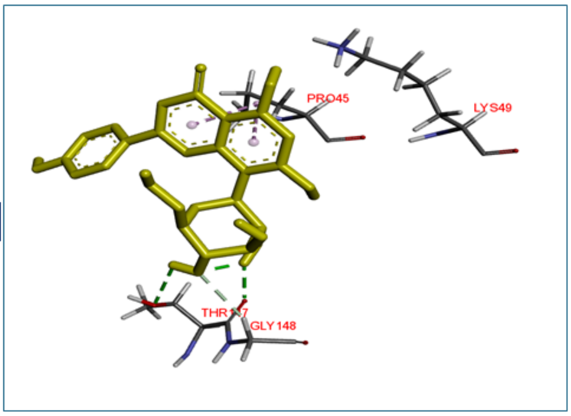

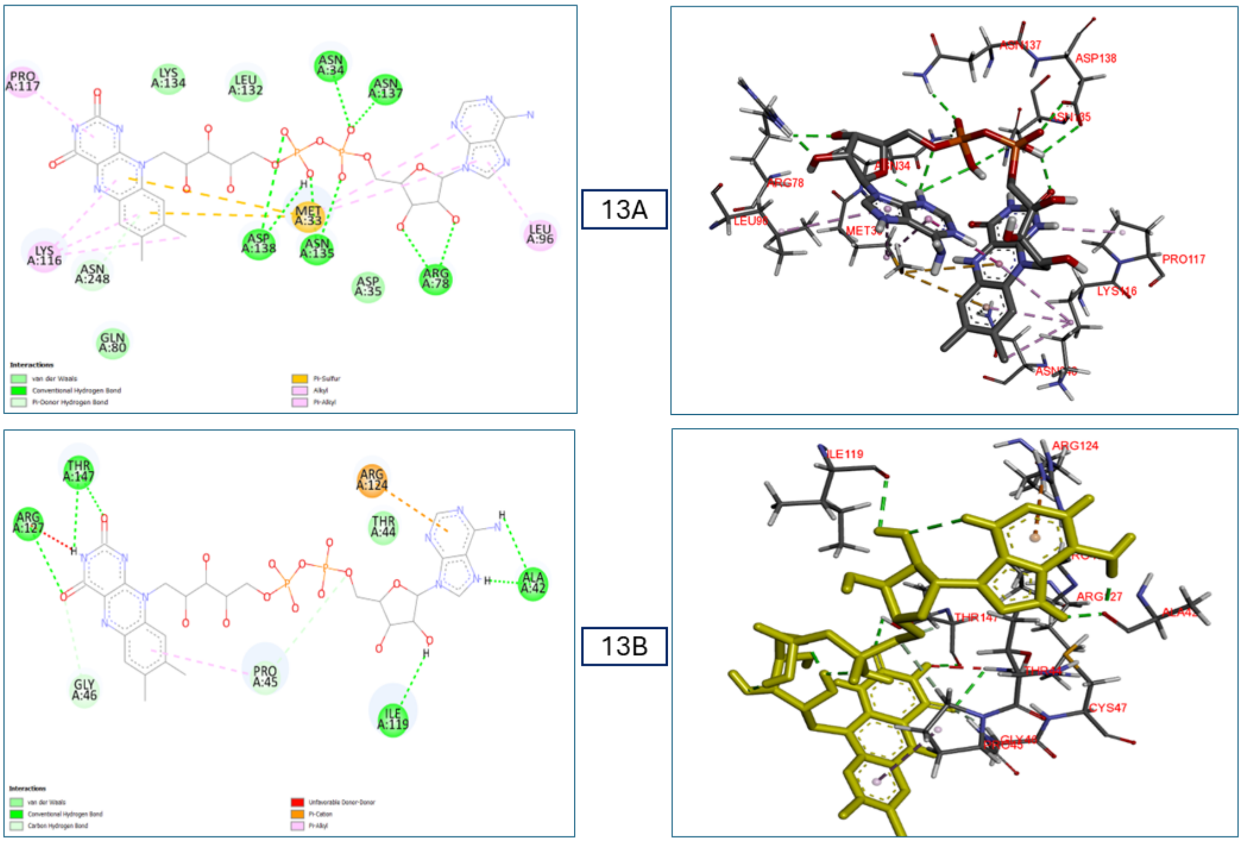

Figure S6. Representative molecular interactions (2D and 3D) of the most dominant chemical compounds (1-12), 1: Gentiobiosylglycerol, 2: Helonioside B, 3: Hydropiperoside, 4: Isoorientin, 5: Isovitexin, 6: Kazinol A, 7: Lapathoside A, 8: Lapathoside B, 9: Orientin, 10: Quercetin-3-*O*-6''-(3-hydroxyl-3-methylglutaryl)-D-glucopyranoside, 11: Vanicoside B, and 12: Vitexin, identified in different *R. vesicarius* organs with **A:** NADPH oxidase (NO) receptor (PDB ID:2CDU, 1.80Å, and **B:** human peroxiredoxin 5 enzyme (PDB code: 1HD2, 1.50 Å), 13: FAD (Flavin-adenine dinucleotide) as a co-crystallized ligand.
